# Health and nutritional aspects of sustainable diet strategies and their association with environmental impacts: a global modelling analysis with country-level detail

**DOI:** 10.1016/S2542-5196(18)30206-7

**Published:** 2018-10

**Authors:** Marco Springmann, Keith Wiebe, Daniel Mason-D'Croz, Timothy B Sulser, Mike Rayner, Peter Scarborough

**Affiliations:** a Oxford Martin Programme on the Future of Food and Centre on Population Approaches for Non-Communicable Disease Prevention, Nuffield Department of Population Health, University of Oxford, Oxford, UK; b Environment and Production Technology Division, International Food Policy Research Institute, Washington, DC, USA; c Global Food and Nutrition Security Group, Commonwealth Scientific and Industrial Research Organisation, St Lucia, QLD, Australia

## Abstract

**Background:**

Sustainable diets are intended to address the increasing health and environmental concerns related to food production and consumption. Although many candidates for sustainable diets have emerged, a consistent and joint environmental and health analysis of these diets has not been done at a regional level. Using an integrated health and environmental modelling framework for more than 150 countries, we examined three different approaches to sustainable diets motivated by environmental, food security, and public health objectives.

**Methods:**

In this global modelling analysis, we combined analyses of nutrient levels, diet-related and weight-related chronic disease mortality, and environmental impacts for more than 150 countries in three sets of diet scenarios. The first set, based on environmental objectives, replaced 25–100% of animal-source foods with plant-based foods. The second set, based on food security objectives, reduced levels of underweight, overweight, and obesity by 25–100%. The third set, based on public health objectives, consisted of four energy-balanced dietary patterns: flexitarian, pescatarian, vegetarian, and vegan. In the nutrient analysis, we calculated nutrient intake and changes in adequacy based on international recommendations and a global dataset of nutrient content and supply. In the health analysis, we estimated changes in mortality using a comparative risk assessment with nine diet and weight-related risk factors. In the environmental analysis, we combined country-specific and food group-specific footprints for greenhouse gas emissions, cropland use, freshwater use, nitrogen application, and phosphorus application to analyse the relationship between the health and environmental impacts of dietary change.

**Findings:**

Following environmental objectives by replacing animal-source foods with plant-based ones was particularly effective in high-income countries for improving nutrient levels, lowering premature mortality (reduction of up to 12% [95% CI 10–13] with complete replacement), and reducing some environmental impacts, in particular greenhouse gas emissions (reductions of up to 84%). However, it also increased freshwater use (increases of up to 16%) and had little effectiveness in countries with low or moderate consumption of animal-source foods. Following food-security objectives by reducing underweight and overweight led to similar reductions in premature mortality (reduction of up to 10% [95% CI 9–11]), and moderately improved nutrient levels. However, it led to only small reductions in environmental impacts at the global level (all impacts changed by <15%), with reduced impacts in high-income and middle-income countries, and increased resource use in low-income countries. Following public health objectives by adopting energy-balanced, low-meat dietary patterns that are in line with available evidence on healthy eating led to an adequate nutrient supply for most nutrients, and large reductions in premature mortality (reduction of 19% [95% CI 18–20] for the flexitarian diet to 22% [18–24] for the vegan diet). It also markedly reduced environmental impacts globally (reducing greenhouse gas emissions by 54–87%, nitrogen application by 23–25%, phosphorus application by 18–21%, cropland use by 8–11%, and freshwater use by 2–11%) and in most regions, except for some environmental domains (cropland use, freshwater use, and phosphorus application) in low-income countries.

**Interpretation:**

Approaches for sustainable diets are context specific and can result in concurrent reductions in environmental and health impacts globally and in most regions, particularly in high-income and middle-income countries, but they can also increase resource use in low-income countries when diets diversify. A public health strategy focused on improving energy balance and dietary changes towards predominantly plant-based diets that are in line with evidence on healthy eating is a suitable approach for sustainable diets. Updating national dietary guidelines to reflect the latest evidence on healthy eating can by itself be important for improving health and reducing environmental impacts and can complement broader and more explicit criteria of sustainability.

**Funding:**

Wellcome Trust, EAT, CGIAR, and British Heart Foundation.

**Research in context**

**Evidence before this study**

Several systematic reviews of the sustainable diet literature have been published since 2014. These reviews have suggested that reductions in environmental impacts of food production are generally proportional to reductions in animal-source foods. However, most studies included in the reviews were national case studies from high-income countries with a predominant focus on greenhouse gas emissions as environmental impact, and the studies generally used different reference diets, environmental footprints, and scenario designs, all of which complicates comparisons between studies. With a few exceptions, the health impacts of the sustainable-diet scenarios were often not explicitly analysed beyond adherence to national dietary guidelines or directional changes in nutrient levels. One systematic review of the health impacts of diets with reduced greenhouse gas emissions found no consistent relationship, but some association of such diets with decreased micronutrient content.

**Added value of this study**

Here, we increase the evidence base beyond summaries of national case studies by using a study design that allows for a consistent and region-specific assessment of the health and environmental impacts of dietary changes for all world regions and more than 150 individual countries. Our study includes a full nutritional analysis, a comparative risk analysis with nine dietary and weight-related risk factors, and an environmental analysis with five domains, including greenhouse gas emissions, cropland use, freshwater use, nitrogen application, and phosphorus application. Our results indicate that energy-balanced, predominantly plant-based dietary patterns that are in line with the current evidence on healthy eating can lead to reductions in environmental impacts in high-income and middle-income countries, while improving nutrient levels and reducing diet-related premature mortality in all regions. In low-income countries, adoption of healthier diets can increase demand for environmental resources, in part due to inefficient production systems and baseline diets high in staple crops. Across regions, the associations between health and environmental benefits are strongest for greenhouse gas emissions, moderate for the demand for cropland, nitrogen, and phosphorus, and small for freshwater use. Our analysis contributes to several open questions in the literature regarding the association between environmental and health impacts, the importance of improving energy imbalances and changing dietary composition, and the generalisability of national case studies.

**Implications of all the available evidence**

Sustainable diets are context specific. They attain nutritional adequacy and reduce diet-related mortality by addressing both dietary composition and energy balance, and they balance environmental impacts between global and regional scales. Our study shows that a synergistic perspective on sustainable diets would include technological and management-related aspects, in addition to dietary and environmental aspects. Updating national dietary guidelines to reflect the latest evidence on healthy eating is important for improving health and reducing environmental impacts, and can complement broader and more explicit criteria of sustainability.

## Introduction

The food people eat impacts their health and the health of the environment. Imbalanced diets low in fruits, vegetables, nuts, and whole grains and high in red and processed meat are responsible for the greatest health burden worldwide and in most regions.^1^ In addition to imbalanced diets, about 2 billion people are overweight and obese, 2 billion have nutritional deficiencies, and about 800 million are still suffering from hunger due to poverty and poorly developed food systems.^2^ As the dietary transition towards more processed and high-value food products (in terms of cost and perceived value) continues in many regions of the world, these dietary health risks are expected to worsen.^3^

The environmental impacts of food production are similarly daunting. Agriculture is responsible for about a quarter of all greenhouse gas emissions,^4^ it occupies about 40% of the Earth's surface^5^ and uses 70% of all freshwater resources,^6^ and the overapplication of fertilisers in some regions has led to pollution of surface water and groundwater and created dead zones in oceans.^7^ As a result, the global food system has contributed to the crossing of several of the proposed planetary boundaries that attempt to define a safe operating space for humanity on a stable Earth system.^8^ In the absence of dedicated mitigation strategies or changes in demand, many of these environmental impacts are expected to intensify as demand for foods with greater environmental impact, such as meat and dairy, increases and the global population grows from 7 billion to a predicted 10 billion in the next 30 years.^9^

The concept of sustainable diets combines the challenges of creating a food system that supplies healthy diets for a growing population while reducing its environmental impacts and staying within planetary boundaries.^10^, ^11^ The literature on sustainable diets has grown substantially in the past decade,^12^, ^13^, ^14^, ^15^ and the concept has been expanded to economic, ethical, and cultural aspects of diets.^10^ However, consistent health analyses of commonly proposed diets are scarce,^16^ and approaches that are based primarily on health rather than environmental objectives are, with few exceptions,^3^, ^17^ rarely considered.

Here, we use a comprehensive modelling framework for more than 150 countries and regions to analyse the environmental and health impacts of three different approaches to sustainable diets, with a focus on the nutritional implications and impacts on chronic, non-communicable disease mortality, and how they relate to changes in greenhouse gas emissions, cropland use, freshwater use, nitrogen application, and phosphorus application. We distinguish between three different approaches: those that follow the environmental objectives of reducing the impacts of animal-source foods; those that follow the food-security objectives of addressing energy imbalances; and those that follow the public health objectives of encouraging nutritionally balanced dietary patterns based on available evidence on healthy eating.

## Methods

### Scenario design

In this global modelling analysis, we assessed the effects of three sets of dietary-change strategies (panel; appendix pp 7–9) on health and environmental factors in more than 150 countries and compare these effects across different regions worldwide.

Panel **Dietary-change strategies for sustainable diets**

**Reduction of animal-source foods following environmental objectives**

Replacement of 25–100% of animal-source foods with plant-based ones at constant total calorie intake (ani-25, ani-50, ani-75, and ani-100); plant-based replacements consist of 75% legumes and 25% fruits and vegetables

**Improving calorie intake and weight levels following food-security objectives**

Improvement of 25–100% in energy imbalances (kcal-25, kcal-50, kcal-75, and kcal-100) with simultaneous reductions in underweight, overweight, and obesity

**Using balanced diet patterns following public health objectives**

Nutritionally balanced diet patterns in line with available evidence on healthy eating:

• Flexitarian: no processed meat, small amounts of red meat (one serving per week), moderate amounts of other animal-source foods (poultry, fish, and dairy), and generous amounts of plant-based foods (fruits, vegetables, legumes, and nuts)
• Pescatarian: replaces meat with two-thirds fish and seafood and a third fruits and vegetables
• Vegetarian: replaces meat with two-thirds legumes and a third fruits and vegetables
• Vegan: replaces all animal-source foods with two-thirds legumes and a third fruits and vegetables

Our baseline data consists of current and projected levels of food consumption and weight distributions. In our main analysis, we focused on the year 2010 for analysing nutrient adequacy and on the year 2030 for the mortality and environmental analyses to allow for a transition time for dietary and technological changes. Additionally, we used the years 2010 and 2050 in sensitivity analyses to study the effects of technological and socioeconomic changes.

We estimated baseline and projected food intake for more than 150 countries by adapting food demand projections from the International Model for Policy Analysis of Agricultural Commodities and Trade (IMPACT) that were based on a harmonised dataset of country-specific food availability data, and we adjusted those for food waste at the household level (appendix pp 2–4).^19^ For estimating the prevalence of underweight (body-mass index [BMI] <18 kg/m^2^), overweight (BMI ≥25 to <30 kg/m^2^), and obesity (BMI ≥30 kg/m^2^) in each country, we fitted log-normal distributions to WHO estimates of mean BMI and the prevalence of overweight and obesity using a cross-entropy method that jointly minimised the deviation of the prevalence data,^20^ and we projected weight changes using correlations between changes in mean BMI and changes in food availability (appendix pp 5–6).^20^

In the first diet pattern set that we assessed, which was based on environmental concerns by reducing animal-source products, we progressively reduced the amount of animal-source foods in each country's diet by 25% (ani-25), 50% (ani-50), 75% (ani-75), and 100% (ani-100) and replaced it with plant-based foods. On the basis of observational data on food-group substitution across dietary patterns, we chose a substitution rule by which two-thirds of animal-source foods were replaced by legumes and a third by fruits and vegetables. We kept the total energy content of the diets constant to isolate the effects of changes in dietary composition in this set of scenarios. In the second set, based on food security and improving energy balance, we progressively reduced levels of underweight, overweight, and obesity in a simultaneous fashion by 25% (kcal-25), 50% (kcal-50), 75% (kcal-75), and 100% (kcal-100). For adjusting total energy intake, we applied scaling factors to baseline diets that preserved their composition. In the third set, based on public health priorities, we constructed four nutritionally balanced dietary patterns that are in line with evidence on healthy eating.^18^ For that purpose, we used energy-balanced varieties of the flexitarian, pescatarian, vegetarian, and vegan dietary patterns defined by the EAT-*Lancet* Commission on Healthy Diets from Sustainable Food Systems (appendix pp 7–9). The flexitarian dietary patterns contain no processed meat, low amounts of red meat (including beef, lamb, and pork) and sugar, moderate amounts of poultry, dairy, and fish, and generous amounts of fruits, vegetables, legumes, and nuts. The other three dietary patterns replace meat (pescatarian or vegetarian) or all animal-source foods (vegan) with two-thirds either fish and seafood (pescatarian diets) or legumes (vegetarian and vegan diets) and a third fruits and vegetables. We regionalised the public health dietary patterns for each country by preserving the national preferences for types of grains, fruits, red meat, and fish. The diet patterns were all compared with a benchmark diet based on our estimates of current and future food consumption and weight distributions, including increased consumption of high-value products (animal-source foods), in line with income and population changes.

### Nutrient analysis

We analysed the nutrient adequacy of the diet scenarios by calculating their nutrient content and comparing these values to international recommendations. For calculating the nutrient content, we paired the consumption of each food group with its nutrient density as reported in the Global Expanded Nutrient Supply dataset,^21^ a global dataset of nutrient supply of 23 nutrients across 225 food categories for more than 150 countries, supplemented by nutritional data on pantothenate and vitamin B12 from the nutrient databases maintained by Harvard University and the US Department of Agriculture. For our analysis, we aggregated the nutrient dataset to the commodity and regional detail of our consumption data, and we normalised calorie densities to those of the UN Food and Agriculture Organization for consistency with our diet scenarios (appendix pp 10–11). We then compared the calculated nutrient content of the diet scenarios to recommendations by WHO. Because the recommendations differ by age and sex, we calculated population-level average values for each nutrient by using the age and sex structure for the year of analysis based on data by the Global Burden of Disease project and forward projections by the UN Population Division. Our estimates of recommended energy intake account for the age-specific and sex-specific energy needs for a moderately active population with US height as an upper bound and include the energy costs of pregnancy and lactation.^22^ Our estimates of calcium intake account for the average calcium content of drinking water in line with previous assessments.^23^ Because WHO did not set guidelines for phosphorus and copper, we used recommended intakes for these nutrients from the US Institute of Medicine.

### Mortality analysis

To analyse the implications of dietary change for chronic disease mortality, we constructed a comparative risk assessment framework with nine risk factors and five disease endpoints. The risk factors included high consumption of red meat, low consumption of fruits, vegetables, nuts and seeds, fish, and legumes, as well as being underweight (BMI <18·5 kg/m^2^), overweight (BMI ≥25 to <30 kg/m^2^), or obese (BMI ≥30 kg/m^2^). The disease endpoints were coronary heart disease, stroke, type 2 diabetes mellitus, cancer (in aggregate and as site-specific ones, such as colon and rectum cancers), and an aggregate of other causes that are associated with changes in weight. The disease endpoints accounted for about half of all deaths in 2015,^24^ and the risk factors were responsible for two-thirds of deaths attributable to dietary risk factors, and for a third of all attributable deaths.^1^

We estimated the mortality and disease burden attributable to dietary risk factors by calculating population impact fractions and applying those to age-specific and country-specific mortality rates (appendix pp 12–13).^25^ Population impact fractions are the proportions of cases of disease that would be avoided when the risk exposure was changed from a baseline situation (the benchmark diet) to a counterfactual situation (the dietary scenarios). We used relative risk estimates from meta-analyses of prospective cohort studies for dietary risks, which relate the risk factors to the disease endpoints, and pooled cohort studies for weight-related risks (appendix pp 14–20). In line with the meta-analyses, we included non-linear dose–response relationships for fruits and vegetables, nuts and seeds, and fish, and assumed linear dose-response relationships for the remaining risk factors. Because our analysis was primarily focused on mortality from chronic diseases, we focused on adults aged 20 years or older, and we adjusted the relative risk estimates for attenuation with age on the basis of a pooled analysis of cohort studies focused on metabolic risk factors^26^ in line with other assessments.^24^ In addition to changes in total mortality, we calculated years of life lost (appendix p 20), but focus on changes in premature mortality among people aged 30–70 years in the main analysis.

### Environmental analysis

For analysing the environmental impacts of the diet scenarios, we used a food-systems model that connects food consumption and production across regions (appendix pp 21–22), and we paired the production estimates with country-specific environmental footprints for greenhouse gas emissions, cropland use, freshwater use, and nitrogen and phosphorus application (appendix pp 22–24).^9^ The food-systems model accounts for trade, feed, and processing of primary commodities and is calibrated with data from the IMPACT agriculture–economic model.^19^ The greenhouse gas emissions associated with agriculture included methane and nitrous oxide emissions, but they exclude carbon dioxide emissions which, following the methods of the International Panel on Climate Change, are allocated to the energy sector or others. Freshwater use, as assessed here, denotes the consumption of surface water and groundwater, and nitrogen and phosphorus application are associated with fertiliser use. The footprints for animal-source foods include the indirect impacts associated with feed production and, for greenhouse gas emissions, direct impacts associated with methane emissions. The projection of environmental footprints includes improvements in technology and management practices along different socioeconomic development pathways (appendix pp 22–25).^9^

In this study, we report on the environmental analysis for contextualisation and focus on the comparison between the health and environmental impacts. For that purpose, we focus on the directional changes in the environmental parameters, which provide a good indication for increased environmental pressures in most regions and globally.^27^ Some exceptions exist, especially for fertiliser use where increased application in low-applying regions can lead to increased agricultural yields without major environmental impacts.^28^, ^29^, ^30^ Our socioeconomic development trajectories include a rebalancing of fertiliser application between overapplying and underapplying regions by 2050,^28^ which reduces the potential for such exceptions.

### Uncertainty analysis

We accounted for the major uncertainties in each analysis. In the comparative risk analysis, we calculated uncertainty intervals associated with changes in mortality using error propagation and the CIs of the relative risk parameters (appendix pp 13, 28, 29). In the nutritional analysis, we explicitly calculated low and high supply values of each nutrient on the basis of the reported CIs (appendix p 26). And in the environmental analysis, we assessed uncertainty by considering different population and income projections, which change the absolute amount and the dietary composition of foods demanded (appendix p 25).

### Role of the funding source

The funder of the study had no role in study design, data collection, data analysis, data interpretation, or writing of the report. All authors had full access to all the data in the study and the corresponding author had final responsibility for the decision to submit for publication.

## Results

**Figure 1:**
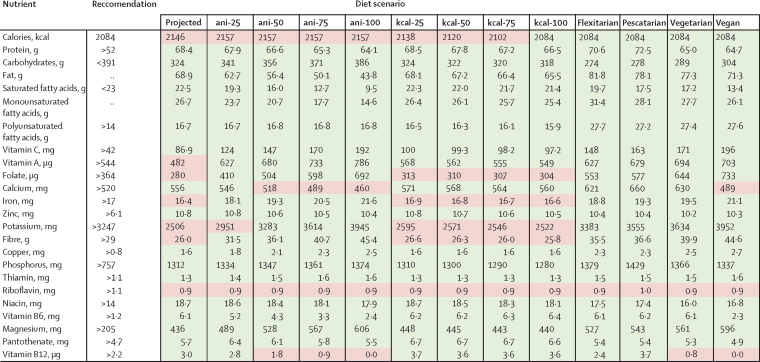
Nutrient supply by diet scenario in 2010 Red values are those that are lower than minimum recommendations or higher than maximum recommendations.

Across the dietary-change scenarios, nutrient availability was generally improved for nutrients that were at low levels at baseline (2010), but with large differences between the scenarios (figure 1; CIs and regional results are reported in the appendix pp 26–27). In the scenarios that replace animal-source foods (ani-25, ani-50, ani-75, and ani-100), the macronutrient content of diets changed towards lower protein and fat content, with large reductions in saturated fatty acids. Protein intake remained adequate in high-income and middle-income countries, but they decreased to lower than recommended amounts in low-income countries. Micronutrient intake improved, particularly in high-income and middle-income countries, where large amounts of animal-source foods could be replaced by plant-based ones. In the high-income and middle-income countries, the baseline low levels of vitamin A, folate, iron, potassium, and fibre increased to greater than recommended values, but calcium, pantothenate (B5), and vitamin B12 decreased to less than recommended levels under full substitution. In low-income countries, the small amounts of animal-source foods that were replaced were not enough to sufficiently increase vitamin A and potassium, and calcium and riboflavin also did not achieve recommended values.

In the scenarios focused on improving energy balance (kcal-25, kcal-50, kcal-75, and kcal-100), total energy intake was reduced to achieve recommended values in high-income and middle-income countries, whereas it was increased to achieve recommendations in low-income countries (appendix p 27). Increased energy intake improved micronutrient intake in low-income countries, but vitamin A, folate, calcium, potassium, and riboflavin remained below recommended values. In high-income and middle-income countries, reductions in energy intake did not improve the baseline low values of folate, iron, potassium, and fibre.

The scenarios based on balanced dietary patterns (flexitarian, pescatarian, vegetarian, and vegan) combined the nutritional impacts of improving energy balance with food-based dietary guidelines for all regions. As a result, most of the nutrients that are at low levels in the baseline diets (vitamin A, folate, iron, potassium, and fibre), increased to recommended values in all four patterns. However, as in the other scenarios based on energy balance or animal-source food substitution, riboflavin remained low, and calcium and vitamin B12 fell below recommended values in the vegetarian or vegan scenarios, or both (appendix p 27). These nutrients would have to be supplemented to attain the recommended value.

**Figure 2:**
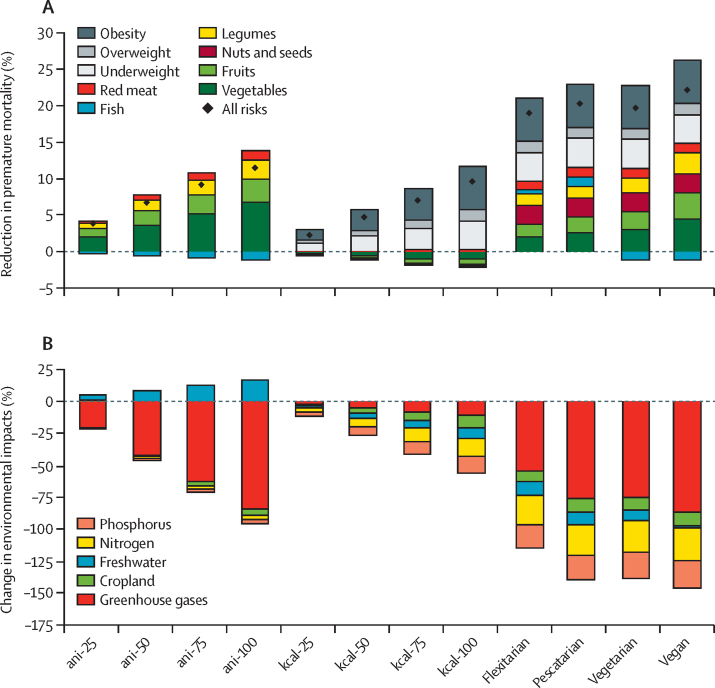
Premature mortality and environmental impacts of diet scenarios in 2030 (A) Diamonds show reductions in premature mortality due to diet patterns and bars show proportion contributions of individual risk factors to the reduction. Total percentage contributions can exceed 100% because individual risks are attenuated when combined and can be compensated by opposing risk factors. (B) Percentage change in environmental impacts.

**Figure 3:**
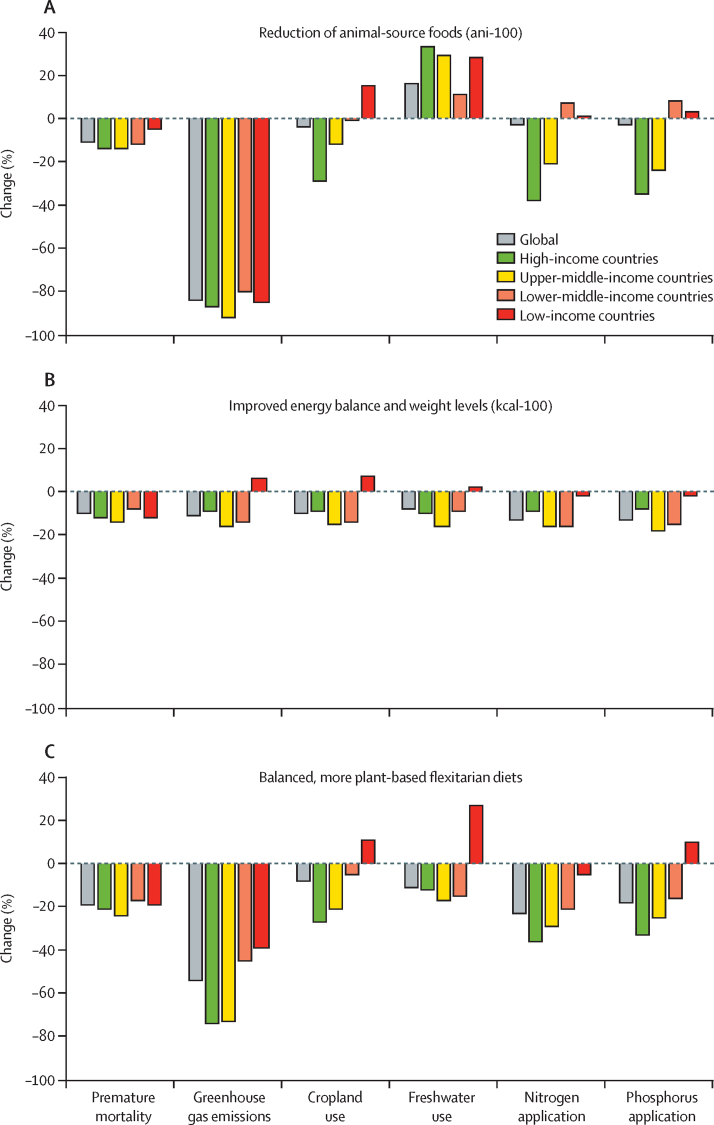
Regional changes in premature mortality and environmental impacts of dietary change The scenarios include diets in which all animal-source foods have been replaced by plant-based ones (ani-100; A), diets with optimal energy intake and weight levels (kcal-100; B), and flexitarian diets that are energy balanced and contain small amounts of animal-source foods (flexitarian; C).

In the comparative risk assessment, premature mortality decreased both with reductions in animal-source foods and with improvements in the energy balance of diets (figure 2A). Progressively replacing animal-source foods with plant-based foods led to progressive reductions in premature mortality of 4% (95% CI 4–4) in the ani-25 scenario up to 12% (10–13) in the ani-100 scenario in 2030 (absolute values are in the appendix pp 28–29). More than half of the total percentage reduction was due to increased vegetable consumption (51–58% across the scenarios), a third of the difference was due to increased fruit consumption (29–31%), a fifth due to increased legume consumption (18–23%), and a tenth due to reductions in red meat consumption (8–11%; figure 2A). Reduction in fish intake led to a 0·3–1% increase in premature mortality across the scenarios. Coronary heart disease (35–36%), stroke (32–34%), and cancer (29–30%) each accounted for about a third of deaths averted, and a small number were from type 2 diabetes (2–3%; appendix p 30). The reductions in premature mortality were two to three times greater in high-income and middle-income countries (12–14% in the ani-100 scenario), where a larger portion of animal-source foods can be substituted, than in low-income countries (5%; figure 3).

Progressively improving the energy balance of diets by reducing both underconsumption and overconsumption, while preserving the overall composition of diets (kcal-25, kcal-50, kcal-75, and kcal-100 scenarios), led to reductions in premature mortality from 2% (95% CI 2–3) in the kcal-25 scenario to 10% (9–11) in the kcal-100 scenario (figure 2A). Across risk factors, reductions in obesity contributed the most to the overall reduction in premature mortality (61–65% across the scenarios), followed by reductions in underweight (42–43%) and reductions in overweight (excluding obesity; 15–17%). As energy intake was adjusted upwards or downwards, the intake of specific foods increased or decreased, and in aggregate led to a 2% increase in mortality, in particular from reductions in fruit and vegetable consumption. Adjusting weight levels without affecting nutrition-sensitive food groups (eg, by adjusting staple foods) would increase the overall reduction in premature mortality by 19% (to a total reduction in premature mortality of 11%). Across disease endpoints, most averted premature deaths were from non-cardiovascular causes related to weight (about 60% with each of the four scenarios), followed by type 2 diabetes (17%), cancer (9–12%), coronary heart disease (9–11%), and stroke (2–3%; appendix p 30). The reductions in premature mortality were generally evenly distributed across regions (8–14% across income groups in the kcal-100 scenario), with the greatest reduction in the upper-middle-income (14%) and high-income (12%) countries, which have high burdens of obesity, followed by low-income countries, (12%) which have high burdens of underweight, and lower-middle-income countries (8%) with an intermediate weight profile (figure 3).

Combining changes in dietary composition with changes in energy balance substantially increased the reductions in premature mortality that each strategy can achieve. Dietary changes to balanced flexitarian, pescatarian, vegetarian, and vegan diets led to reductions in premature mortality of 19% (95% CI 18–20) for the flexitarian scenario to 22% (18–24) for the vegan scenario (figure 2A). Reductions in underweight, overweight, and obesity contributed similar proportions to reductions in premature mortality (11% across the four diets), as did change in diet composition (9–12%). In the vegetarian and vegan scenarios, small increases in premature mortality (about 1%) from reduced fish intake were compensated (in part in the vegetarian scenario, and in full in the vegan scenario) by reductions in premature mortality from additional intake of legumes, fruits, and vegetables. Increases in nut consumption in the four scenarios, an aspect not included in the other dietary-change strategies, led to reductions in premature mortality (about 3%) in addition to those associated with replacing animal-source foods. Across disease endpoints, the averted premature deaths were about a quarter each from coronary heart disease (25–29%) and non-cardiovascular causes (25–30%), a fifth (19–21%) from cancer, followed by stroke (14–18%) and type 2 diabetes (8–10%; appendix p 30). The reductions in premature mortality were generally evenly distributed across regions (eg, 19–24% across income groups in the flexitarian scenario), with the greatest reductions in upper-middle-income countries, where diets and energy intake were most imbalanced (figure 3).

For all 12 of the dietary-change approaches, the changes in environmental impacts differed by environmental domain and region. Progressively replacing animal products with plant-based foods led to large reductions in greenhouse gas emissions (from 20% in the ani-25 scenario to 84% in the ani-100 scenario) because the demand for emissions-intensive animal-source foods was reduced (figure 2B). However, freshwater use was increased (from 4% in the ani-25 scenario to 16% in the ani-100 scenario) because the demand for water-demanding crops, such as legumes, vegetables, and fruits was increased (figure 2B; appendix pp 31–32). Regional analyses showed further differences. In upper-middle-income and high-income countries, replacement of animal-source foods led to reduced cropland use (12% in upper-middle-income countries and 29% in high-income countries), nitrogen application (22% and 38%), and phosphorus application (25% and 35%), in line with reductions in the demand for livestock-related intensive feed production and fertilisation (figure 3). By contrast, these impacts increased in low-income and lower-middle-income countries (cropland 1% in lower-middle-income countries and 15% in low-income countries; nitrogen application 7% and 1%; and phosphorus application 7% and 3%), which use less intensive feeds and fertilisers and had generally lower yields, so that the impacts of increased demand for legumes and vegetables outweighed feed-related reductions (figure 3).

Improving the energy balance of diets led to moderate reductions across environmental impacts globally (8–13% in the kcal-100 scenario; figure 2B; appendix pp 31–32). Environmental impacts were reduced in high-income and middle-income countries (8–18% in the kcal-100 scenario), where levels of overweight and obesity required a reduction in energy intake. By contrast, impacts were increased (3–8% in the kcal-100 scenario) for greenhouse gas emissions, cropland use, and freshwater use in low-income countries, where levels of underweight required an increase in energy intake (figure 3).

Globally, moving to the balanced dietary patterns resulted in large reductions in greenhouse gas emissions (54–87% across the scenarios), medium-level reductions in nitrogen application (23–25%) and phosphorus application (18–21%), and small to moderate reductions in cropland (8–11%) and freshwater use (2–11%; figure 2B; appendix pp 31–32). Greenhouse gas emissions and nitrogen application were reduced in all regions, but the changes in cropland use, freshwater use, and phosphorus application were split into reductions in high-income and middle-income countries and increases in low-income countries (figure 3; appendix p 31), in line with regional differences in yields, water use, and fertilisation intensity.

**Figure 4:**
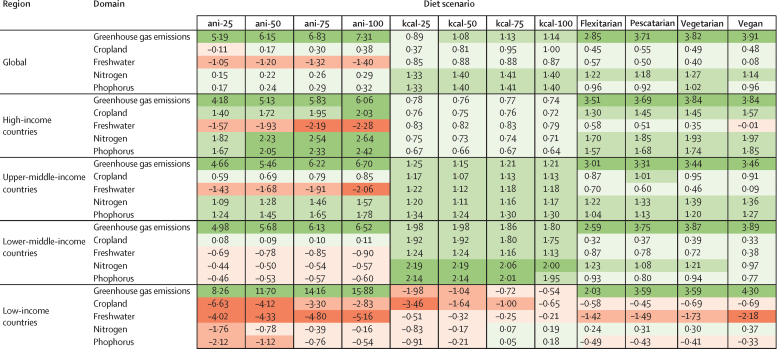
Coefficients of association between health and environmental impacts Coefficients were calculated by dividing the percentage changes in environmental impacts by the percentage changes in premature mortality. Positive values (green) indicate that health and environmental changes are aligned (larger values show stronger positive associations), whereas negative values (red) indicate opposing changes (more-negative values show stronger negative associations). Darker shades show stronger associations, whereas lighter shades show weaker associations.

Several scenarios showed alignment between the health and environmental benefits of dietary change, but large differences existed between regions and dietary-change approaches (Figure 3, Figure 4). For the scenarios reducing animal-source foods (ani scenarios), reductions in premature mortality were positively associated with reductions in greenhouse gas emissions and negatively associated with freshwater use. Other environmental domains had mostly positive relationships with mortality in high-income and upper-middle-income countries, and negative relationships in low-income and lower-middle-income countries. For the scenarios aimed at improving energy balance (kcal scenarios), the changes in environmental impacts were positively associated with changes in premature mortality in high-income and middle-income countries, but they were negatively associated in low-income countries (figure 4). The balanced dietary-pattern scenarios (flexitarian, pescatarian, vegetarian, and vegan) showed the greatest alignment of health and environmental impacts, with positive association for almost all environmental domains in high-income and middle-income countries, but negative associations for cropland use, freshwater use, and phosphorus application in low-income countries.

In a sensitivity analysis, we analysed the influence the time period has on the health and environmental impacts of dietary change. Analysing dietary changes in 2050 instead of 2030 resulted in greater alignment of the health and environmental impacts as technologies and management practices improved, particularly for freshwater and cropland use (appendix p 33). Conversely, analysing dietary changes in 2010 resulted in greater relative increases in cropland and freshwater use than in 2030. Considering data of the emissions of carbon dioxide (based on a global meta-analysis of lifecycle analyses^31^) instead of the data limited to methane and nitrous oxide emissions as in the main analysis, increased greenhouse gas emissions, particularly from fishing, and as a result widened the difference between the pescatarian and vegetarian scenarios (appendix p 34).

## Discussion

The concept of sustainable diets combines health and environmental concerns. Although many candidates for sustainable diets have emerged, no consistent and joint environmental and health analysis of these diets has been done at a regionally comparative level. Here, we examined three stylised approaches to sustainable diets using an integrated health and environmental modelling framework for more than 150 countries. Our results show that a public health approach focused on dietary changes towards predominantly plant-based diets that are in line with evidence on healthy eating performs better in reducing environmental pressures, potential nutrient deficiencies, and diet-related mortality than approaches motivated only by environmental and food-security concerns.

Our analysis has several strengths. It significantly improves on the methods used in existing global assessments by doubling the number of risk factors and environmental indicators covered.^3^, ^17^ It improves the detail of dietary scenarios by including, in the balanced dietary-pattern scenarios, recommended ranges for all major food groups based on the available evidence on healthy eating as reviewed by the EAT-*Lancet* Commission on Healthy Diets from Sustainable Food Systems. And it covers all major countries and regions in a comparative fashion, which allows for the identification of regional differences that extend the evidence base beyond national case studies.^12^, ^14^, ^15^

Where comparisons are possible, our results are consistent with empirical data and other estimates with similar coverage. The nutritional estimates are based on existing datasets, and the assessment of nutritional levels and potential deficiencies is in line with existing analyses for most nutrients, but can differ for calcium for which we used global recommended intake values instead of the sometimes higher national values,^32^ and for zinc, for which we assumed medium bioavailability rather than using a function of absorption that is not yet validated.^23^ Our mortality estimates from changes in dietary risk factors are similar to those of comparable dietary patterns in cohort studies.^33^, ^34^ However, by analysing the disease burden of dietary patterns in equilibrium, we abstracted from many real-world complexities, such as time lags between adoption of diets and changes in mortality. Although most of the relative risk factors used have been adjusted for major confounding factors, such as smoking, bodyweight, and other dietary risks, residual confounding with other parameters cannot be ruled out completely. In line with our joint focus on the health and environmental impacts of dietary change, we focused on those risk factors that we could include on the basis of food-availability data, and we did not include risk factors, such as processed meat and whole grains, that would have required processing factors on the basis of data derived by a different method.^35^ Including those risk factors as part of recommendations to reduce processed meat consumption and increase whole grain consumption would further increase the estimated benefits of dietary change in the dietary-pattern and substitution scenarios (appendix pp 14–20). The environmental assessment is based on data that reflects current environmental impacts at the country level (appendix pp 22–23), and the scenario changes are in line with other assessments with similar detail.^9^

Our results highlight the regional differences in the health and environmental impacts of dietary-change strategies. Following an environmental strategy by substituting animal-source foods can be particularly effective in high-income countries for improving nutrient levels, lowering premature mortality, and reducing some environmental impacts, in particular greenhouse gas emissions. However, it can also lead to increased freshwater use, and has little effectiveness in countries with low or moderate consumption of animal-source foods. Following a food-security strategy by improving the energy balance of diets can lead to similar reductions in premature mortality, but in our model scenarios it only moderately improved nutrient levels and led to small reductions in environmental impacts at the global level, with reduced impacts in high-income and middle-income countries, and increased resource use in low-income countries. Following a public health strategy by adopting energy-balanced, low-meat dietary patterns that are in line with available evidence on healthy eating addressed the problems of high regional variability of the animal-substitution scenarios and the low nutritional and environmental effectiveness of the weight scenarios. Adopting the balanced and predominantly plant-based dietary patterns led to an adequate nutrient supply, except for a small number of nutrients (riboflavin, calcium, and vitamin B12), which might have to be supplemented, large reductions in premature mortality, and significant reductions in environmental impacts globally and in most regions, except for some environmental domains (cropland use, freshwater use, and phosphorus application) in low-income countries.

Our analysis has several implications for the study of sustainable diets. First, qualitative differences exist between the health and environmental benefits that can be attained by dietary changes. Our analysis suggests that although a comprehensive and context-specific public health strategy for dietary change can lead to healthier diets in all regions, differences between the environmental impacts and regions are large. Dietary changes towards healthy, low-meat diets can be effective in reducing greenhouse gas emissions, moderately effective in reducing cropland use and fertiliser application in high-income and middle-income countries, but less effective for reducing freshwater use, particularly in low-income countries. Changes in low-income countries depend more strongly on technological improvements and changes in management,^9^ suggesting that a synergistic perspective on sustainable diets should include both technological and dietary aspects. Although reducing greenhouse gas emissions is important at the global level for mitigating climate change, changes in the other domains relate to predominantly local environmental impacts. This highlights the need for context-specific strategies that balance environmental impacts between global and regional scales.

Second, addressing dietary composition and energy intake as part of food-based dietary guidelines could be a comprehensive strategy for achieving sustainable diets. Whether food-based dietary guidelines should include sustainability criteria has been a major issue of discussion in several countries.^13^, ^36^ In this study, we find that when food-based dietary recommendations reflect available evidence on healthy eating, including balanced energy intake, low amounts of red meat and sugar, low to moderate amounts of other animal-source foods, and generous amounts of fruits, vegetables, legumes, and nuts, then the resulting diets would be in line with sustainability criteria of reducing environmental impacts in most regions, and they would still improve dietary health in the remaining ones. However, many national dietary guidelines do not reflect this evidence on healthy eating and include no or too lax limits for animal-source foods, particularly meat and dairy,^37^ despite an opposing evidence base (appendix pp 14–20).^38^, ^39^, ^40^ A general problem is that many nutrient recommendations used to formulate food-based dietary guidelines are based on a few short-term studies with few participants that measure nutrient pass-through in high-consuming individuals instead of lower limits.^41^ By contrast, most of the available evidence on healthy eating comes from long-term and large-scale epidemiological cohort studies.^18^ Our results show that updating national dietary guidelines to reflect the latest evidence on healthy eating can by itself be important for improving health and environmental sustainability, and can complement broader and more explicit criteria of sustainability.

Some unanswered questions remain. We were not able to include all aspects of importance to sustainable diets, including biodiversity impacts and economic aspects.^10^ Although biodiversity impacts are related to land use in a given region, impacts can be expected to differ on the basis of a region's biodiversity richness and how land use is managed. With respect to economic aspects, large changes in food demand and supply can be expected to affect production methods, technologies, and commodity prices, which in turn would impact environmental footprints, agricultural incomes, the affordability of diets, and purchasing behaviour. Identifying concrete policy options that could support the dietary changes modelled here is similarly important. Globally, overweight and obesity, as well as consumption of red meat and dairy are projected to increase in the future without dedicated policy approaches.^42^, ^43^ Although informational campaigns and voluntary actions by industry can be important, the literature on behavioural change suggests that they are unlikely to be effective on their own.^44^, ^45^ Instead, crosscutting regulatory approaches that focus on the whole food environment,^46^ combine multiple incentives including fiscal ones,^47^, ^48^ and offer support and positive re-enforcement for individuals^49^, ^50^ have been successful in specific contexts, but would need to be upscaled to lead to substantial dietary changes at the population level. Finding effective combinations of policies and approaches that consider local characteristics will be essential for successfully upscaling initiatives and achieving reductions in the health and environmental burden at the population level and globally.

## Data sharing
