# Supplementary material for: Health and nutritional aspects of sustainable diet strategies and their association with environmental impacts: a global modelling analysis with country-level detail
Source: Lancet Planet Health. 2018 Oct;2(10):e451–61. doi: 10.1016/S2542-5196(18)30206-7 (PMC6182055; doi:10.1016/S2542-5196(18)30206-7)
Supplement: Supplementary appendix [file mmc1.pdf]

THE LANCET
Planetary Health

Supplementary appendix

This appendix formed part of the original submission and has been peer reviewed. We post it as supplied by the authors.

Supplement to: Springmann M, Wiebe K, Mason-D'Croz D, Sulser T, Rayner M, Scarborough P. Health and nutritional aspects of sustainable diet strategies and their association with environmental impacts: a global modelling analysis with country-level detail. *Lancet Planet Health* 2018; **2:** e451–61.

The health and nutritional aspects of sustainable diet strategies and their relationship to environmental impacts – a comparative global modelling analysis with country-level detail

Appendix

Table of Contents

A.1 Supplementary data 2
A.1.1 Consumption estimates 2
A.1.2 Weight estimation 5
A.1.3 Diet scenarios 7
A.2 Supplementary methods 10
A.2.1 Nutrient analysis 10
A.2.2 Health analysis 12
A.2.3 Relative risk parameters 14
A.2.4 Environmental analysis 21
A.3 Supplementary results 26
References 35

A.1 Supplementary data

A.1.1 Consumption estimates

Our baseline data consists of current and projected levels of food consumption and weight distributions. Estimates of food consumption are based on a harmonised dataset of country-specific food availability data, adjusted for food waste at the household level.[1,2] We adapted the food availability data for current and future years from the International Model for Policy Analysis of Agricultural Commodities and Trade (IMPACT), a global agriculture-economic model which uses economic, water, and crop models to simulate global food production, consumption, and trade of 62 agricultural commodities for over 150 world regions.[1] Its demand projections account for changes in income and population as drivers, in line with other projections.[3,4]

For the dietary risk assessment, we converted the food availability estimates into food consumption estimates by using regional data on food wastage at the consumption level, combined with conversion factors into edible matter[2]. Supplementary Table 2 lists the waste percentages and conversion factors used. No conversion factor was used for red meat, because the waste percentages reported in Supplementary Table 1 were obtained for carcass weight (including bone), and therefore included wastage of non-edible parts.

The full regional aggregation used in this study is listed in Supplementary Table 2, and an overview of food-consumption estimates for current and future years are provided in Supplementary Table 3.

Supplementary Table 1. Waste percentages at consumption according to FAO[2]

Food items	Europe	USA, Canada, Oceania	Industri-alized Asia	Sub-Saharan Africa	North Africa, West and Central Asia	South and Southeast Asia	Latin America
Cereals	0.25	0.27	0.2	0.01	0.12	0.03	0.1
Roots and tubers	0.17	0.3	0.1	0.02	0.06	0.03	0.04
Oilseeds and pulses	0.04	0.04	0.04	0.01	0.02	0.01	0.02
Fruits and vegetables	0.19	0.28	0.15	0.05	0.12	0.07	0.1
Meat	0.11	0.11	0.08	0.02	0.08	0.04	0.06
Milk	0.07	0.15	0.05	0.001	0.02	0.01	0.04

Conversion factors into edible matter: 0.82 for roots, 0.79 for maize, 0.78 for wheat, 1 for rice, 0.78 for other grains, 0.77 for fruits and vegetables, 1 for meat, 1 for oilseeds and pulses, 1 for milk

Supplementary Table 2. Regional aggregation

**High-income countries (HIC)**		
Australia	Hungary	Portugal
Austria	Iceland	Republic of Korea
Belgium and Luxembourg	Ireland	Rest of Arab Peninsula
Canada	Israel	Saudi Arabia
Croatia	Italy	Slovakia
Cyprus	Japan	Slovenia
Czech Republic	Netherlands	Spain
Denmark	New Zealand	Sweden
Finland	Norway	Switzerland
France	Other Caribbean	United Kingdom
Germany	Other Southeast Asia	United States of America
Greece	Poland	
**Upper middle-income countries (UMC)**		
Botswana	Dominican Republic	Baltic States
Algeria	Jamaica	Kazakhstan
Gabon	Mexico	Other Balkans
Namibia	Panama	Romania
South Africa	Peru	Russian Federation
Argentina	Uruguay	Fiji
Brazil	Venezuela (Bolivarian Republic of)	Malaysia
Chile	Lebanon	Other Pacific Ocean
Colombia	Libya	
Costa Rica	Bulgaria	
Cuba	Belarus	
**Lower middle-income countries (LMC)**		
Angola	Paraguay	Turkmenistan
Côte d'Ivoire	El Salvador	Ukraine
Cameroon	Djibouti	Bhutan
Lesotho	Egypt	Indonesia
Nigeria	Iran (Islamic Republic of)	India
Other Atlantic Ocean	Jordan	Sri Lanka
Other Indian Ocean	Pakistan	Thailand
Swaziland	Sudan	Timor-Leste
Belize	Syrian Arab Republic	China
Bolivia (Plurinational State of)	Tunisia	Mongolia
Ecuador	Albania	Philippines
Guyanas South America	Armenia	Papua New Guinea
Guatemala	Azerbaijan	
Honduras	Georgia	
Nicaragua	Republic of Moldova	
**Low-income countries (LIC)**		
Burundi	Mali	Afghanistan
Benin	Mozambique	Yemen
Burkina Faso	Mauritania	Kyrgyzstan
Central African Republic	Malawi	Tajikistan
Congo	Niger	Uzbekistan
Eritrea	Senegal	Bangladesh
Ethiopia	Sierra Leone	Myanmar
Ghana	Chad	Nepal
Guinea	Togo	Cambodia
Gambia	United Republic of Tanzania	Lao People's Democratic Republic
Guinea-Bissau	Uganda	Solomon Islands
Kenya	Zambia	Viet Nam
Liberia	Zimbabwe	
Madagascar	Haiti	

Supplementary Table 3. Food consumption (g/d) by food group, region, and year. Regions include high-income countries (HIC), upper middle-income countries (UMC), lower middle-income countries (LMC), low-income countries (LIC), and an aggregate of all countries (Global). Years include 2010, 2030, and 2050.

Food groups	2010					2030					2050				
	Global	HIC	UMC	LMC	LIC	Global	HIC	UMC	LMC	LIC	Global	HIC	UMC	LMC	LIC
wheat	117.6	135.8	154.8	121.1	56.6	122.7	140.2	158.6	129.2	64.5	126.0	144.8	161.7	134.2	70.4
rice	126.4	34.5	48.7	158.4	170.2	122.5	32.9	50.0	152.0	152.9	116.6	31.2	49.5	145.0	137.0
maize	33.0	16.8	69.6	21.9	58.3	37.4	17.1	73.2	25.4	66.2	40.2	17.2	73.7	27.5	70.7
other grains	21.8	10.7	11.8	21.2	42.4	26.0	10.7	11.4	24.2	53.6	31.6	10.8	11.4	28.2	66.8
roots	133.8	108.6	135.0	125.2	192.9	146.3	105.6	129.6	139.1	215.1	153.0	104.4	124.2	145.7	224.7
legumes	16.7	9.2	22.8	15.6	24.8	20.3	9.9	25.9	19.0	30.2	24.1	10.5	28.2	22.3	37.2
soybeans	4.8	3.6	2.4	6.5	1.8	7.0	3.6	2.7	10.3	2.0	6.2	3.4	2.7	9.2	2.2
nuts and seeds	13.3	11.5	12.7	14.6	11.6	15.0	12.1	13.4	16.6	13.8	15.2	12.6	13.3	16.2	15.8
vegetables	229.1	204.3	161.6	291.5	86.4	281.9	224.7	187.3	373.9	108.4	327.8	229.8	198.6	450.5	137.0
temperate fruits	36.8	74.3	41.2	31.2	18.9	42.0	77.7	45.4	39.2	22.7	44.9	81.5	49.0	42.0	27.8
tropical fruits	62.3	77.0	86.9	61.9	25.0	74.0	83.3	99.8	77.1	36.5	82.9	87.4	108.5	86.7	52.3
starchy fruits	28.3	15.5	36.5	24.7	48.1	39.8	16.8	43.0	34.1	74.7	52.7	17.7	47.6	41.5	111.5
sugar	51.4	68.9	96.1	43.1	22.5	62.7	71.6	107.2	60.6	28.1	71.6	74.5	114.7	73.8	35.0
palm oil	6.4	3.9	5.4	7.8	5.3	10.1	4.5	7.0	13.2	7.5	12.9	4.9	8.6	16.8	10.7
vegetable oils	21.6	46.4	29.6	15.9	9.7	22.0	44.8	31.1	17.6	10.8	22.5	45.5	32.9	18.0	12.6
beef	25.2	60.1	52.9	11.8	12.9	29.7	62.1	57.9	18.1	18.3	33.2	64.8	61.1	20.7	27.9
lamb	5.3	5.1	4.4	5.9	4.4	7.2	5.7	5.6	8.2	6.4	9.1	6.8	6.7	9.9	10.2
pork	37.9	78.2	25.4	36.2	10.7	37.9	76.2	27.5	37.6	12.9	35.9	76.7	29.1	34.0	15.0
poultry	30.7	71.2	58.9	18.5	7.9	40.2	82.2	74.9	30.0	12.1	47.1	89.4	84.9	38.7	16.7
eggs	21.7	32.2	25.7	22.4	4.1	23.2	31.0	27.1	25.8	5.4	23.2	30.8	28.1	26.1	6.8
milk	221.7	515.3	328.4	153.8	80.3	253.0	520.7	341.9	214.5	91.6	263.9	528.3	356.3	232.5	109.4
shellfish	5.8	10.6	2.4	6.5	1.1	6.4	10.8	3.2	7.5	1.2	6.5	11.3	3.7	7.6	1.4
freshwater fish	7.7	3.7	2.9	9.7	8.5	9.8	4.1	4.1	12.1	11.2	11.6	4.3	4.8	13.4	16.0
pelagic fish	3.2	6.4	4.9	2.4	1.2	2.4	5.1	4.2	2.0	0.6	2.0	4.5	3.8	1.8	0.4
demersal fish	4.9	10.6	4.5	3.7	3.5	4.3	9.1	4.8	3.3	3.4	3.9	8.3	5.8	3.2	3.3
other ctops	12.5	30.5	15.6	8.7	6.1	13.4	31.3	17.6	10.0	7.1	14.1	31.7	18.7	10.9	8.5

A.1.2 Weight estimation

For the weight-related risk assessment, we estimated changes in weight as shifts in the baseline weight distribution by using the historical relationship between national food availability and mean BMI. We estimated the baseline distribution by fitting a log-normal distribution to WHO estimates of mean BMI and the prevalence of overweight and obesity using a cross-entropy method[5]. Cross-entropy estimation is a Bayesian technique for recovering parameters and data which have been observed imperfectly. The cross-entropy approach redefines the estimation problem as estimating and minimizing the divergence from the original prior while satisfying various constraints. In our application, we take mean BMI values as given and use the cross-entropy method to find the shape and position parameters of the log-normal distribution which jointly minimize the deviation of the estimates of the prevalence of overweight and the prevalence of obesity from the input parameters.

We estimated the relationship between national food availability and mean BMI by pairing FAO food availability data for the years 1980-2009 with WHO data on mean BMI for the same period. Using a polynomial trend yielded the following relationship ($R^2 = 0.46$):

$$BMI(r) = (-9.53 \cdot 10^{-7}) \cdot kcal(r)^2 + (7.87 \cdot 10^{-3}) \cdot kcal(r) + 10.18$$

where $kcal(r)$ denotes food availability in region r in terms of kcal per person per day, and $BMI(r)$ denotes the average mean BMI in that region. Supplementary Figure 1 provides a graphical depiction.

Based on the relationship between mean BMI and food availability, we estimated the changes in the weight distribution as follows. We calculated the mean BMI values for the years 2010 and 2030 using food availability projections from the IMPACT model, and we then used the percentage change in mean BMI between 2010 and 2030 to shift the baseline BMI distribution. In shifting the weight distribution, we held constant the distribution's shape parameter, $\sigma(r)$, and re-calculated its position parameter $\mu(r)$ based on the estimated mean: $\mu(r) = \log BMI(r) - \frac{\sigma(r)^2}{2}$. Analyses were conducted to assess the impact of holding the shape parameter constant, which showed that results were not sensitive to this assumption. Supplementary Table 4 provides an overview of our baseline estimates of current and future weight distributions.

Supplementary Figure 1. Association between food availability and mean BMI based on data from FAO and WHO for the years 1980-2009.

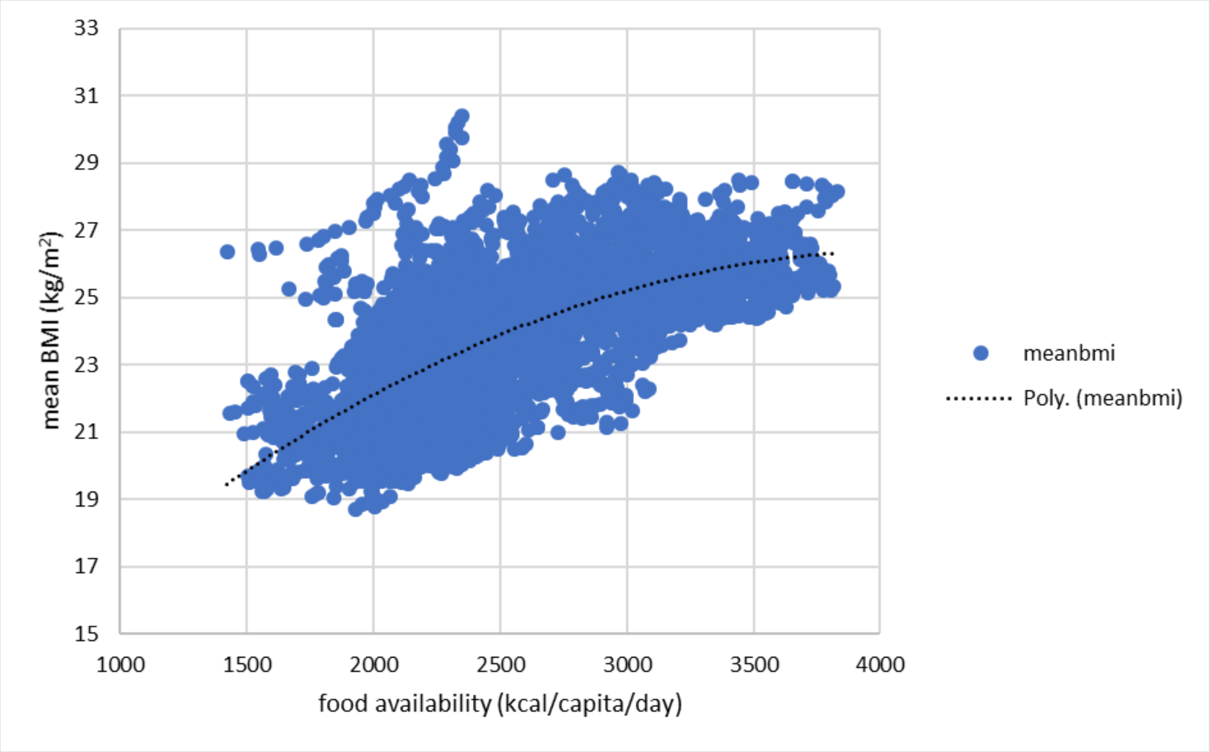

Supplementary Table 4. Prevalence of underweight, normal weight, overweight, and obesity by year and region.

Year	Region	underweight	normal	overweight	obesity
2010	Global	0.13	0.51	0.25	0.11
2010	HIC	0.05	0.40	0.32	0.23
2010	UMC	0.05	0.38	0.34	0.23
2010	LMC	0.14	0.55	0.23	0.08
2010	LIC	0.23	0.55	0.17	0.05
2030	Global	0.10	0.49	0.28	0.13
2030	HIC	0.04	0.39	0.33	0.24
2030	UMC	0.04	0.36	0.34	0.25
2030	LMC	0.11	0.53	0.27	0.10
2030	LIC	0.18	0.53	0.22	0.07
2050	Global	0.09	0.47	0.29	0.15
2050	HIC	0.04	0.38	0.33	0.25
2050	UMC	0.04	0.35	0.35	0.27
2050	LMC	0.09	0.51	0.28	0.12
2050	LIC	0.14	0.50	0.25	0.10

A.1.3 Diet scenarios

We defined three sets of scenarios. In the first set (kcal-25, kcal-50, kcal-75, kcal-100), we progressively reduced levels of underweight, overweight and obesity in a simultaneous fashion by 25%, 50%, 75% and 100%. In the second set (ani-25, ani-50, ani-75, ani-100), we progressively reduced the amount of animal source foods in each country's diet by 25%, 50%, 75% and 100% and substituted it with plant-based foods. In the third set (FLX, PSC, VEG, VGN), we constructed four nutritionally balanced dietary patterns that are in line with the current evidence on healthy eating.[6–8]

For the latter, we adopted energy-balanced varieties of the flexitarian, pescatarian, vegetarian, and vegan dietary patterns defined by the EAT-Lancet Commission on Healthy Diets from Sustainable Food Systems. Estimates of energy balances were based on the calorie needs of a moderately active population of US characteristics for height divided into 5-year age groups [9], something that can be seen as an upper bound. Calorie needs reach a maximum of 2500 kcal/d for ages 20-24 (averaged between men and women), but are reduced to 2000 kcal for ages 65 and older. Supplementary Table 5 provides an overview. The average calorie needs differed by region based on its age composition, and ranged around 2100 kcal/d.

Supplementary Table 5. Calorie needs (kcal/d) by age and sex.

Age	Female	Male	Average
0-4	1200	1200	1200
5-9	1520	1600	1560
10-14	1920	2120	2020
15-19	2040	2760	2400
20-24	2200	2800	2500
25-29	2000	2600	2300
30-34	2000	2600	2300
35-39	2000	2600	2300
40-44	2000	2600	2300
45-49	2000	2400	2200
50-54	1800	2400	2100
55-59	1800	2400	2100
60-64	1800	2400	2000
65-69	1800	2200	2000
70-74	1800	2200	2000
75-79	1800	2200	2000
80-84	1800	2200	2000
85-89	1800	2200	2000
90-94	1800	2200	2000
95-99	1800	2200	2000
100+	1800	2200	2000

The flexitarian diets (FLX) included at least 500 g/d of fruits and vegetables of different colours and groups (the composition of which is determined by regional preferences), at least 100 g/d of plant-based protein sources (legumes, soybeans, nuts), modest amounts of animal-based proteins, such as poultry, fish, milk, and eggs, and limited amounts of red meat (1 portion per week), refined sugar (<5% of total energy), vegetable oils that are high in saturated fat (in particular palm oil), and starchy foods which have a relatively high glycaemic

index. Supplementary Table 6 provides an overview of the food-based recommendations used for constructing the flexitarian-diet scenario.

Supplementary Table 6. Food-based dietary recommendations for healthy, more plant-based (flexitarian) diets. The recommendations include recommended minimum (min) and maximum (max) intake expressed by weight or calories, and servings. Fish and seafood can be substituted by plant-based foods (legumes, soybeans, nuts and seeds, fruits and vegetables) in vegetarian diets.

Food item	minimum level		maximum level	
	g/d	serving	g/d	serving
wheat<br>rice<br>maize<br>other grains<br>roots			860 kcal/d for energy balance	3-4 (1/3 of energy)
legumes	50	1/2		
soybeans	25	1/4		
nuts & seeds	50	2		
vegetables	300	3-4		
fruits	200	2-3		
sugar			31	5% of energy
palm oil			6.8	1
vegetable oil			80	1/3 of energy
beef<br>lamb<br>pork			14	1/7
poultry			29	1/2
eggs			13	1/5
milk			250	1
shellfish<br>fish (freshwater)<br>fish (demersal)<br>fish (pelagic)	28	1/2		

Based on the flexitarian diets, we constructed more specialised diets, including pescatarian, vegetarian and vegan diets, which are in line with dietary guidelines and observed dietary patterns in specialised cohorts [10,11]. For the pescatarian diets (PSC), meat-based protein sources in the flexitarian diets were replaced (on a kcal basis) to two thirds by fish and seafood, and one third by fruits and vegetables; for the vegetarian diets (VGT), they were replaced to two thirds by plant-based proteins, and one third by fruits and vegetables; and for the vegan diets (VGN), all animal-based protein sources were replaced to two thirds by plant proteins, and one third by fruits and vegetables. We aimed to preserve the regional character of dietary patterns by maintaining the regional composition of specific foods within broader categories, such as preferences for specific staple crops (wheat, maize, rice, etc) and fruits (temperate, tropical). Supplementary Table 7 provides an overview of all diet scenarios included in the analysis.

Supplementary Table 7. Food consumption in diet scenarios in 2010 (food groups in g/d, and total energy intake in kcal/d).

Food group	Diet scenarios												
	BMK	FLX	PSC	VEG	VGN	ani-25	ani-50	ani-75	ani-100	kcal-25	kcal-50	kcal-75	kcal-100
total energy	2156	2083	2083	2083	2083	2156	2156	2156	2156	2138	2120	2101	2083
wheat	118	91	91	91	91	118	118	118	118	117	116	115	115
rice	126	81	81	81	81	126	126	126	126	125	125	124	123
maize	33	23	23	23	23	33	33	33	33	33	33	32	32
other grains	22	15	15	15	15	22	22	22	22	22	22	22	22
roots	134	100	100	100	100	137	137	137	137	136	135	134	133
legumes	17	50	50	62	78	30	43	56	69	17	17	17	18
soybeans	5	25	25	31	35	11	17	23	29	5	5	5	5
nuts and seeds	13	51	51	51	51	13	13	13	13	13	13	13	13
vegetables	229	355	397	424	495	301	373	445	517	226	223	220	217
temperate fruits	37	61	68	73	87	51	64	78	91	37	36	36	35
tropical fruits	62	101	114	123	149	85	107	129	151	62	61	60	59
starchy fruits	28	40	40	40	40	31	31	31	31	31	31	31	31
sugar	51	27	27	27	27	51	51	51	51	51	50	50	50
palm oil	6	4	4	4	4	6	6	6	6	6	6	6	6
vegetable oil	22	42	42	42	42	22	22	22	22	21	21	21	20
beef	25	5	0	0	0	19	13	6	0	25	24	24	24
lamb	5	2	0	0	0	4	3	1	0	5	5	5	5
pork	38	5	0	0	0	28	19	9	0	37	36	36	35
poultry	31	19	0	0	0	23	15	8	0	30	29	29	28
eggs	22	10	10	10	0	16	11	5	0	21	21	21	20
milk	222	155	155	155	0	167	111	56	0	221	219	218	216
shellfish	6	7	15	0	0	4	3	1	0	6	6	6	5
freshwater fish	8	14	26	0	0	6	4	2	0	8	7	7	7
pelagic fish	3	5	10	0	0	2	2	1	0	3	3	3	3
demersal fish	5	7	15	0	0	4	2	1	0	5	5	5	5

A.2 Supplementary methods

A.2.1 Nutrient analysis

We estimated the nutrient content of foods by pairing the consumption of each food group with its nutrient density as reported in the Global Expanded Nutrient Supply (GENuS) dataset, a global dataset of nutrient supply of 23 nutrients across 225 food categories for over 150 countries,[12] supplemented by nutritional data on pantothenate and vitamin B12 from the nutrient databases maintained by Harvard University (Harvard T.H. Chan School of Public Health Nutrition Department's Food Composition Tables) and the US Department of Agriculture (USDA Food Composition Database). For our analysis, we aggregated the nutrient dataset to the commodity and regional detail of our consumption data, and we normalised calorie densities to those of the Food and Agriculture Organization for consistency with our diet scenarios. Supplementary Table 8 provides an overview of the nutrient contents used in the analysis.

We compared the calculated nutrient content of the diet scenarios to recommendations of the World Health Organization (WHO).[13,14] Because the recommendations differ by age and sex, we calculated population-level average values for each nutrient by using the age and sex structure for the year of analysis based on data by the Global Burden of Disease project and forward projections by the Population Division of the United Nations.[15,16] Our estimates of recommended energy intake take into account the age and sex-specific energy needs for a moderately active population of US height as an upper bound,[9,17] and include the energy costs of pregnancy and lactation.[17] Our estimates of calcium intake take into account the average calcium content of drinking water, in line with previous assessments.[18] Because the WHO did not set guidelines for phosphorus and copper, we adopted their recommended intakes from the US Institute of Medicine.

Supplementary Table 8. Nutrient content of food groups (global average). Units are kcal/g for calories; g/g for protein, fat, carbohydrates, fibre, saturated fatty acids, mono-unsaturated fatty acids, and poly-unsaturated fatty acids; microgram/g for vitamin, folate, and vitaminB12; and mg/g for all others.

Food group	calories	protein	carbohydrates	fat	saturatedFA	monounsatFA	polyunsatFA	vitamin C	vitamin A	folate	calcium	iron	zinc	potassium	fiber	copper	sodium	phosphorus	thiamin	riboflavin	niacin	vitamin B6	magnesium	pantothenate	vitamin B12
wheat	2.96	0.11	0.62	0.02	0.00	0.00	0.01	0.00	0.00	0.35	0.33	0.04	0.02	2.82	0.05	0.00	0.09	2.88	0.00	0.00	0.05	0.01	1.17	0.01	
rice	3.67	0.07	0.82	0.01	0.00	0.00	0.00			0.08	0.15	0.01	0.01	0.91	0.02	0.00	0.06	1.22	0.00	0.00	0.03	0.00	0.43	0.01	
maize	3.06	0.08	0.61	0.04	0.01	0.01	0.02		0.20	0.19	0.11	0.02	0.02	2.54	0.07	0.00	0.09	2.20	0.00	0.00	0.02	0.00	0.97		
other grains	3.02	0.10	0.62	0.03	0.01	0.01	0.01	0.00	0.00	0.23	0.36	0.06	0.02	2.71	0.08	0.01	0.08	2.51	0.00	0.00	0.03	0.01	1.26	0.00	
roots	0.85	0.01	0.19	0.00	0.00	0.00	0.00	0.17	0.10	0.16	0.16	0.01	0.00	3.31	0.02	0.00	0.10	0.45	0.00	0.00	0.01	0.00	0.19	0.00	
legumes	3.58	0.23	0.60	0.02	0.00	0.00	0.01	0.02	0.05	3.37	1.33	0.07	0.04	10.55	0.13	0.01	0.22	3.72	0.00	0.00	0.03	0.00	1.53	0.01	
soybeans	3.54	0.31	0.26	0.15	0.03	0.04	0.10	0.05	0.24	3.33	1.89	0.07	0.03	15.35	0.09	0.01	0.04	5.98	0.00	0.00	0.02	0.00	2.47	0.01	
nuts and seeds	3.44	0.13	0.13	0.27	0.03	0.14	0.09	0.02	0.02	0.93	0.57	0.03	0.02	4.51	0.05	0.01	0.08	2.58	0.00	0.00	0.05	0.00	1.27	0.01	
vegetables	0.26	0.01	0.05	0.00	0.00	0.00	0.00	0.17	0.64	0.28	0.22	0.01	0.00	1.97	0.01	0.00	0.13	0.35	0.00	0.00	0.01	0.00	0.16		
vegetables (dark green)	0.26	0.02	0.04	0.00	0.00	0.00	0.00	0.36	0.93	0.93	0.50	0.01	0.00	2.60	0.02	0.00	0.41	0.38	0.00	0.00	0.00	0.00	0.40	0.00	
vegetables (orange)	0.26	0.01	0.05	0.00	0.00	0.00	0.00	0.18	1.89	0.20	0.20	0.01	0.00	2.80	0.02	0.00	0.17	0.31	0.00	0.00	0.01	0.00	0.14	0.00	
vegetables (other)	0.26	0.01	0.05	0.00	0.00	0.00	0.00	0.13	0.11	0.16	0.17	0.00	0.00	1.52	0.01	0.00	0.05	0.36	0.00	0.00	0.01	0.00	0.11	0.00	
fruits (temperate)	0.45	0.00	0.11	0.00	0.00	0.00	0.00	0.08	0.07	0.04	0.07	0.00	0.00	1.26	0.02	0.00	0.01	0.14	0.00	0.00	0.00	0.00	0.06	0.00	
fruits (tropical)	0.40	0.01	0.09	0.00	0.00	0.00	0.00	0.24	0.35	0.16	0.18	0.00	0.00	1.59	0.02	0.00	0.03	0.15	0.00	0.00	0.00	0.00	0.11	0.00	
fruits (starchy)	0.77	0.01	0.18	0.00	0.00	0.00	0.00	0.08	0.11	0.15	0.07	0.00	0.00	2.83	0.01	0.00	0.01	0.21	0.00	0.00	0.00	0.00	0.22	0.00	
sugar	3.57	0.00	0.89								0.08	0.00	0.00	0.20		0.00	0.03	0.01		0.00			0.01		
palm oil	8.81			0.99	0.52	0.34	0.08		17.04			0.01	0.00				0.00								
vegetable oils	8.81		0.00	0.99	0.16	0.36	0.40		0.00		0.01	0.00	0.00	0.00			0.01	0.00		0.00					
beef	1.64	0.14	0.00	0.12	0.06	0.06	0.01	0.00	0.02	0.05	0.08	0.02	0.03	2.14		0.00	0.48	1.24	0.00	0.00	0.03	0.00	0.13	0.00	0.02
lamb	2.07	0.21		0.22	0.05	0.07	0.01		0.10	0.16	0.68	0.08	0.07	3.75		0.00	0.91	1.94	0.00	0.00	0.06	0.00	0.26		0.02
pork	2.91	0.11	0.01	0.27	0.10	0.12	0.03	0.00	0.08	0.03	0.09	0.01	0.02	2.02		0.00	0.42	1.23	0.00	0.00	0.03	0.00	0.10	0.01	0.01
poultry	1.44	0.14	0.00	0.16	0.06	0.11	0.02	0.01	0.20	0.05	0.08	0.02	0.01	1.68		0.00	0.52	1.20	0.00	0.00	0.04	0.00	0.15	0.01	0.00
eggs	1.43	0.13	0.02	0.09	0.03	0.04	0.02		1.89	0.48	0.55	0.02	0.01	1.34		0.00	1.35	1.94	0.00	0.00	0.00	0.00	0.12	0.02	0.01
milk	0.58	0.04	0.02	0.04	0.03	0.01	0.00	0.00	0.36	0.04	1.26	0.00	0.00	0.57		0.00	0.53	0.75	0.00	0.00	0.00	0.01	0.10	0.00	0.00
shellfish	0.78	0.14	0.02	0.01	0.00	0.00	0.00	0.02	0.35	0.18	1.28	0.05	0.03	2.15		0.00	3.20	2.12	0.00	0.00	0.02	0.00	0.29	0.00	0.07
fish (freshwater)	1.29	0.21	0.01	0.04	0.01	0.02	0.01	0.01	0.29	0.17	0.84	0.01	0.01	3.72		0.00	0.62	2.39	0.00	0.00	0.03	0.00	0.34	0.01	0.03
fish (pelagic)	1.59	0.24	0.00	0.06	0.02	0.02	0.01	0.02	0.33	0.10	0.50	0.01	0.01	4.60		0.00	0.93	2.80	0.00	0.00	0.08	0.00	0.38	0.01	0.10
fish (demersal)	1.02	0.20	0.01	0.02	0.00	0.00	0.00	0.02	0.23	0.11	0.60	0.01	0.01	3.65		0.00	1.19	2.20	0.00	0.00	0.03	0.00	0.31	0.00	0.01
other crops	2.27	0.03	0.18	0.02	0.01	0.01	0.00	0.02	0.78	0.21	0.66	0.03	0.01	3.11	0.04	0.00	0.31	1.04	0.00	0.00	0.02	0.00	0.66	0.13	

A.2.2 Health analysis

We estimated the mortality and disease burden attributable to dietary and weight-related risk factors by calculating population impact fractions (PIFs) which represent the proportions of disease cases that would be avoided when the risk exposure was changed from a baseline situation to a counterfactual situation. For calculating PIFs, we used the general formula[19–21]:

$$PIF = \frac{\int RR(x)P(x)dx - \int RR(x)P'(x)dx}{\int RR(x)P(x)dx}$$

where $RR(x)$ is the relative risk of disease for risk factor level x, $P(x)$ is the number of people in the population with risk factor level x in the baseline scenario, and $P'(x)$ is the number of people in the population with risk factor level x in the counterfactual scenario. We assumed that changes in relative risks follow a dose-response relationship[20], and that PIFs combine multiplicatively[20,22], i.e. $PIF = 1 - \prod_i(1 - PIF_i)$ where the *i*'s denote independent risk factors.

The number of avoided deaths due to the change in risk exposure of risk *i*, $\Delta deaths_i$, was calculated by multiplying the associated PIF by disease-specific death rates, DR, and by the number of people alive within a population, P:

$$\Delta deaths_i(r, a, d) = PIF_i(r, d) \cdot DR(r, a, d) \cdot P(r, a)$$

where PIFs are differentiated by region *r* and disease/cause of death *d*; the death rates are differentiated by region, age group *a*, and disease; the population groups are differentiated by region and age group; and the change in the number of deaths is differentiated by region, age group and disease.

In addition to changes in mortality, we also calculated the years of life saved (YLS) due to a change in dietary and weight-related risk factors. For calculating YLS, we multiplied each age-specific death by the life expectancy expected at that age using the Global Burden of Disease standard abridged life table[22].

We used publicly available data sources to parameterize the comparative risk analysis. Mortality data were adopted from the Global Burden of Disease project[23], and projected forward by using data from the UN Population Division[16]. The relative risk estimates that relate the risk factors to the disease endpoints were adopted from meta-analyses of prospective cohort studies for dietary risks,[24–31] and a pooled cohort study for weight-related risks.[32] In line with the meta-analyses, we included non-linear dose-response relationships for fruits and vegetables,[26] nuts and seeds,[25] and fish,[31] and assumed linear dose-response relationships for the remaining risk factors.[24,27–30] The weight-related relative risk parameters were aggregated to the BMI categories used in this study and normalized to a risk-neutral normal weight category consistent with the epidemiological evidence[32,33]. As our analysis was primarily focused on mortality from chronic diseases, we focused on adults aged 20 year

or older, and we adjusted the relative-risk estimates for attenuation with age based on a pooled analysis of cohort studies focussed on metabolic risk factors,[34] in line with other assessments.[21,35] Supplementary Table 9 provides an overview of the relative-risk parameters used, and the following section provides additional detail.

Supplementary Table 9. Relative risk parameters (mean and low and high values of 95% confidence intervals) per 100g serving for dietary risks and change in weight class for weight-related risks.

Risk factor	Stats	Coronary heart disease	Stroke	Total Cancer	Type-2 diabetes	Colorectal cancer	Other
fruits	mean	0.95	0.77	0.94			
	low	0.92	0.70	0.91			
	high	0.99	0.84	0.97			
vegetables	mean	0.87	0.95	0.94			
	low	0.84	0.90	0.92			
	high	0.90	1.01	0.95			
nuts and seeds	mean	0.84		0.92			
	low	0.82		0.90			
	high	0.86		0.95			
legumes	mean	0.77					
	low	0.65					
	high	0.90					
red meat	mean		1.10		1.14	1.15	
	low		1.05		1.04	1.07	
	high		1.15		1.24	1.24	
fish	mean	0.66					
	low	0.50					
	high	0.87					
underweight	mean	0.68	1.03	1.11			1.75
	low	0.65	0.71	0.94			1.50
	high	0.70	1.47	1.32			2.05
normal	mean						
	low						
	high						
overweight	mean	1.31	1.07	1.10	1.54		0.96
	low	1.24	0.73	1.04	1.42		0.89
	high	1.39	1.59	1.17	1.68		1.03
obese	mean	1.78	1.55	1.40	7.37		1.33
	low	1.64	1.14	1.30	5.16		1.22
	high	1.92	2.11	1.50	10.47		1.46

For the different diet scenarios, we calculated uncertainty intervals associated with changes in mortality based on standard methods of error propagation and the confidence intervals of the relative risk parameters. For the error propagation, we approximated the error distribution of the relative risks by a normal distribution and used that side of deviations from the mean which was largest. This method leads to conservative and potentially larger uncertainty intervals as probabilistic methods, such as Monte Carlo sampling, but it has significant computational advantages, and is justified for the magnitude of errors dealt with here (<50%) (see e.g. IPCC Uncertainty Guidelines).

A.2.3 Relative risk parameters

Dietary risk factors

Dietary risks are the leading risk factors for death globally and in most regions.[20] The Global Burden of Disease Study included 14 different components as dietary risks, such as not eating enough fruit, nuts and seeds, vegetables, whole grains, and omega-3s and eating too much red and processed meat. Dietary factors have been associated with the development of cardiovascular diseases, diabetes, and various cancers, and total mortality.

In this study, we focused on changes in the consumption of total red meat, fish, fruits, vegetables, nuts, and legumes. These risk factors were responsible for two thirds of deaths attributable to dietary risk factors in 2015, and for a third of all attributable deaths in that year.[21] We restricted the selection of relative risk parameters to meta-analyses and pooled prospective cohort studies, which we describe below. In addition to the risk factors included in our analysis, we also reviewed the evidence for other risks, such as white meat, dairy, and whole grains, which we include here for completion and reference.

Red and processed meat

In meta-analyses, the consumption of processed meat, including processed beef, pork, and poultry, has been associated with increased risk of coronary heart disease [36], stroke [28,30,36–38], type 2 diabetes [29,30,36], cardiovascular diseases in general [39,40], site-specific cancers [41–44], total cancer [40], and all-cause mortality [39,40,45].

The association between unprocessed red meat and disease risk is generally weaker, but statistically significant for several disease endpoints. In meta-analyses, the consumption of red meat, including beef and pork, has been associated with increased risk of stroke [28,37,38], type 2 diabetes [29], cardiovascular diseases in general [39], site-specific cancers [27,41–44], and mortality from all causes (including from CVD and cancer) in high-consuming populations [45] and in high-quality studies with long follow-up time [39,40].

There are several plausible explanations for the elevated risks in meat consumers, which support the observational evidence [46]. Mediating factors that are associated with adverse health effects include the composition of dietary fatty acids and cholesterol in red and processed meat, haem iron, as well as sodium, nitrates and nitrites, and advanced glycation end products (AGEs) in processed meats.

For total red meat, we adopted linear dose-response relationships between increased intake and increased risk for stroke, type-2 diabetes, and colorectal cancer from meta-analyses of cohort studies by Chen, Feskens, and Chan and colleagues.[27,29,47] The summary relative-risk estimates per 100 g/d increase in total red meat intake was 1.10 (95% CI, 1.05-1.15; n=4) for stroke, 1.15 (95% CI, 1.07-1.24; n=14) for type-2 diabetes, and 1.14 (95% CI, 1.04-1.24) for colorectal cancer.

White meat

The elevated risks for processed meat also applies to processed white meats, such as processed poultry (and fish). However, the disease associations for unprocessed white meats are less clear. When compared to the baseline diet, there does not seem to be a significant increase in disease risk [39], but substituting other sources of protein with white meat could confer health benefits or detriments, depending on the source of protein that is substituted [48–51]. There are no meta-analyses available that focussed on changes in relative risk from changes in protein sources, but several individual cohort studies provide some guidance. Those indicate that the risk for CHD [49], stroke [48], type 2 diabetes [51] and total mortality [50] can, in part, be reduced for replacement of animal proteins, such as red and processed meat, dairy, poultry, and fish by plant-based protein sources, such as nuts, legumes, and whole grains, but uncertainty intervals were large due to low consumption levels of some of foods.

Dairy

Meta-analyses of prospective cohort studies found no evidence for an association between milk and dairy consumption and mortality from all causes, CHD, and stroke [52–54]. A modest inverse association between milk intake and overall CVD risk was reported by Soedama-Muthu and colleagues [54], but that association was not visible in subgroup analyses, and not replicated in later meta-analyses. Instead, several inconsistencies of that earlier analysis, e.g., with respect to study selection have been identified.[53] Some meta-analyses suggested that milk consumption could reduce the risk of colon cancer [55] and type 2 diabetes [56], but the associations became not statistically significant in each case when adjusted for red and processed meat consumption [55,56]. On the other hand, there is evidence that milk consumption might lead to increased risk of prostate cancer [44,57,58] due to an associated between dairy and insulin-like growth factor 1, an anabolic hormone linked to prostate and other cancers.

Several factors complicate the interpretation of meta-analyses of the health associations of dairy consumption. Three general problems for dairy-related meta-analyses are high heterogeneity of results across individual cohort studies [52,59,60], high degree of potential confounding with other food groups, such as fruits and vegetables and red meat [55,56], and potential conflict of interest in several meta-analyses that were conducted by researchers who received funding from the dairy industry [54,60,61].

It should be noted that milk and dairy consumption is recommended by many nutritional guidelines for meeting nutrient requirements, in particular for calcium. However, the evidence base for such recommendations has been questioned [6], and meta-analyses of randomised controlled trials [62] and observational studies [63] of calcium intake and fracture found no evidence that increasing calcium intake from dietary sources prevents fracture (see also [64]). In addition, lactase persistence, i.e., the ability to digest the milk sugar lactose in adult age, is only present in about a quarter of the world's population, in particular in those from Northern European and Mediterranean decent. The majority of the world's population

(70-75%) lose the ability to digest lactose after weaning, which can lead to gastrointestinal symptoms, such as flatulence, bloating, cramps, and diarrhea upon consumption in some individuals [65–67]. Although lactose intolerance can be managed in a way that milk and dairy products can be consumed in certain quantities [68], the literature reviewed above does not present a strong case for recommending milk and dairy consumption on health grounds.

Seafood

In meta-analyses of prospective cohort studies, low and moderate consumption of fish has been weakly associated with reduced risk of CHD [31,69], stroke [70,71], mortality from all causes [72], and type 2 diabetes which was mediated by location and fish type [73,74]. For most endpoints, risk reduction of mortality reached a lowest point at or below one serving per day (60-80 g/d), and then levelled off (or turned negative) [72].

Several mechanisms have been suggested to explain the moderate health-protective effect of fish consumption. Fish contains omega-3 fatty acids which have been suggested to lower the risk of all-cause mortality and CHD [72]. Multiple mechanisms of omega 3 fatty acids might be involved, including cell growth inhibition and enhanced apoptosis, suppression of neoplastic transformation and antiangiogenicity. In addition, oily fish contains vitamin D which has been suggested to lower the risk of type 2 diabetes.

With regards to the beneficial impacts of omega-3 fatty acids, a pooled analyses of cohort studies [75] confirmed that an increase in the intake of omega-3 fatty acids is associated with reduced risk of mortality from coronary heart disease, and they also showed that plant-derived omega fatty acids have a similar health benefit as fish-derived fatty acids, which indicates that either source is beneficial and can be substituted.

Subgroup and sensitivity analyses conducted in the meta-analyses of fish consumption and disease risk have highlighted additional aspects, in particular cooking methods and substitution effects. In subgroup analyses, several meta-analyses [71–73] found no statistically significant risk reduction with increased fish consumption in Western countries that consume fish predominantly in fried form, compared to significant risk reductions in Asian countries that consume fish boiled or raw. This finding indicates that cooking methods may play a role in risk mediation. In addition, substitution effects can play a role as fish replaces relatively more unhealthy food groups, such as red and processed meat. The sensitivity analysis by Zhao and colleagues [72] indicated that the statistical significant association between fish consumption and reduction in mortality becomes non-significant if studies adjusted for intakes of red meat, and of fruit and vegetables.

For fish, we adopted a non-linear dose-response relationship between increased intake and reduced risk for CHD from a meta-analysis of cohort studies by Zheng and colleagues.[31] The summary relative-risk estimates per 15 g/d increase in fish intake was 0.94 (95% CI, 0.90-0.98; n=17), with no evidence for further reduction beyond an intake of 50 g/d.

Nuts

In meta-analysis of prospective cohort studies, the consumption of nuts has been associated with reduced risk of CHD [25,76–78], type 2 diabetes by reducing body weight [25,76,77], cardiovascular disease in general [25,77–79], cancer [25,79], mortality from respiratory disease, diabetes, and infections [25], and death from all causes [25,77–79], but not from stroke [25,76,78,80,81]. Most of the reduction in risk was observed for an intake of up to six servings (of 28 g) per week (or 15–20 g/d) for most of the outcomes [25].

The suggested mechanism for the risk reduction from nut consumption includes the fat composition of nuts with low proportions of saturated fatty acids, and high proportions of mono-unsaturated and poly-unsaturared fatty acids which have beneficial effects on inflammation, lipid biomarkers, and blood pressure. Nuts are also a good source of biomarkers which are each associated with reductions in CVD risk, such as folate, antioxidant vitamins and compounds, plant sterols, CA, Mg, and K(7).

For nuts, we adopted non-linear dose-response relationships between increased intake and reduced risk for CHD, type-2 diabetes, and cancer from a meta-analysis of 20 cohort studies by Aune and colleagues.[25] The summary relative-risk estimates per 28 grams/day increase in nut intake were 0.71 (95% CI, 0.63-0.80; n=11) for CHD, 0.61 (95% CI, 0.43-0.88; n=4) for type-2 diabetes, and 0.85 (95% CI, 0.76-0.94; n=8) for cancer. Most of the reduction in risk was observed up to an intake of 15-20 g/d.

Legumes

Less meta-analyses have been conducted about the health associations of changes in the consumption of legumes. Legumes are rich in protein, complex carbohydrates, fiber, and various micronutrients, which could lead to positive health impacts. In one meta-analyses, legume consumption was inversely associated with CHD, but not significantly associated with stroke or diabetes [76]. Another meta-analysis found associations between legume consumption and reduced risk of colorectal cancer [82].

For legumes, we adopted a linear dose-response relationship between increased intake and reduced risk for CHD from a meta-analysis of cohort studies by Afshin and colleagues [76]. The summary relative-risk estimate per 4 weekly 100-g servings was 0.86 (95% CI, 0.78-0.94; n=5).

Fruit and vegetables

In meta-analyses, the consumption of fruits and vegetable has been associated with reduced risk of coronary heart disease [26,83–85], stroke [26,85–87], type 2 diabetes in particular for green leafy vegetables [88,89], cardiovascular disease in general [26,90], mortality from all causes [26,91], and modest reductions in total cancer [26] with greater reductions for site-specific cancers [44,92]. Earlier analyses suggested a threshold of five servings per day above which risks are not

reduced further [91], but a recent meta-analyses that included a greater number of studies observed reductions in risk for up to ten servings of fruits and vegetables per day (800 g/d) [26].

Suggested mechanisms include the antioxidant properties of fruits and vegetables that neutralize reactive oxygen species and reduce DNA damage, modulation of hormone metabolism, as well as the benefits from fibre intake on cholesterol, blood pressure and inflammation. Benefits have not been reproducible with equivalent amounts of representative vitamin, mineral and fibre supplements [93,94], which suggests that the micronutrients, phytochemicals, and fibre found in fruits and vegetables act synergistically and through several biological mechanisms to reduce the risk of chronic disease and premature mortality [95,96].

For fruits and vegetable consumption, we adopted non-linear dose-response relationships between increased intake and reduced risk for CHD, stroke, and cancer from a meta-analysis of 95 cohort studies by Aune and colleagues.[26] The summary relative-risk estimates per 200 grams/day were 0.90 (95% CI, 0.86-0.94; n=26) for fruits and CHD, 0.84 (95% CI, 0.79-0.90; n=23) for vegetables and CHD; 0.82 (95% CI, 0.74-0.90; n=19) for fruits and stroke, 0.87 (95% CI, 0.79-0.96; n=14) for vegetables and stroke; 0.96 (95% CI, 0.94-0.99; n=25) for fruits and total cancer, 0.96 (95% CI, 0.93-0.99; n=19) for vegetables and total cancer. For fruits and vegetables combined, the lowest risk for total cancer was observed at an intake of 550-600 g/d, and for CHD and stroke, the lowest risk was observed at 800 g/d, which was at the high end of the range of intake across studies.

Root and tubers

Roots and tubers, such as potatoes and cassava, are the energy stores of plants. In health analyses, they are often not classified as vegetables due to their high starch content and comparatively lower content of vitamins, minerals, and phytochemicals [44], and together with starchy fruits, such as bananas and plantains, are considered a separate category. Although roots and tubers do not appear to have similarly beneficial health impacts as non-starchy fruits and vegetables, there is inconsistent evidence from meta-analyses that roots and tubers are detrimental for health per se, or whether it is the added fats in Western-style consumption patterns, such French fries, that contribute to observed negative health impacts [97–99].

Grains

The health impacts of grain consumption depend on the degree of processing. Milling whole grains to refined grains removes the germ and ban from the endosperm. Whole grains, but not refined grains, have been associated in meta-analyses with reduced risk of cardiovascular disease [100,101], coronary heart disease [100,102], cancer (Aune et al., 2016b), type 2 diabetes [100,103], and other causes of death (Aune et al., 2016b). Their consumption has also been associated with reductions in overweight and obesity (Ye et al., 2012). For most outcomes, risk reductions have been observed for intakes up to seven and a half servings of 30 g each (210-225 g/d in total) (Aune et al., 2016b).

Suggested mechanism refer to the fibre content of whole grains which reduces glucose and insulin responses, lowers concentration of total and low density lipoprotein (LDL) cholersterol, improves the functional properties of the digestive tract (binding, removing, excretion), and decreases inflammatory markers (Aune et al., 2016b).

The consumption of refined grains has, in most cases, not been consistently associated with disease outcomes in meta-analyses [100,104,105], but replacement of refined grains with whole grains would confer reductions in the risks of cardiovascular disease, cancer, and type 2 diabetes as reviewed above.

Sugar

In meta-analyses of prospective cohort studies and randomised controlled trials, the consumption of free (added) sugars and sugar sweetened beverages has been associated with weight gain [106,107] and metabolic syndrome, a cluster of cardio-metabolic risk factors that are predictive of CVD [108,109]. In meta-analyses of prospective cohort studies, sugar sweetened beverages in particular were also associated with increased risk of type 2 diabetes independent of weight gain [110]. Increased risk of type 2 diabetes was also observed for artificially sweetened beverages and fruit juice, but study quality was judged to be low in each case (Imamura et al., 2015).

The underlying mechanisms that have been suggested include incomplete compensation for liquid calories from sugar sweetened beverages, and a high glycemic load from free sugars, both of which lead to weight gain (Malik et al., 2013; Malik and Hu, 2015). Increased diabetes and cardiovascular disease risk also occur independently of weight through adverse glycemic effects and increased fructose metabolism in the liver [111].

Weight-related risk factors

Excess weight is an established risk factor for several causes of death, including ischaemic heart disease,[112,113] stroke,[113–115] and various cancers.[44,116–118] Plausible biological explanations[32,119,120] and the identification of mediating factors[32,121] suggest that the association between body weight and mortality is not merely statistical association, but a causal link independent of other factors, such as diet and exercise.[122–126]

We inferred the parameters describing relative mortality risk due to weight categories from two large, pooled analyses of prospective cohort studies.[32,33] We concentrated on four broad causes of death: ischaemic/coronary heart disease, stroke, cancers, and all other causes. We adopted the relative risks for ischaemic heart disease and stroke from the Prospective Studies Collaboration,[32] which analysed the association between BMI and mortality among 900,000 persons in 57 prospective studies that were primarily designed to evaluate risk factors for cardiovascular disease; and we adopted the relative risks for cancer and all other causes from Berrington de Gonzalez and colleagues,[33] who examined the relationship between BMI and

mortality in a pooled analysis of 19 prospective studies which included 1.46 million adults and which were predominantly designed to study cancer.

From each study, we adopted the relative risk rates for lifelong non-smokers to minimize confounding and reverse causality, and, to increase comparability, we normalized the relative-risk schedule to the lowest risk which, in each case corresponded to a body-mass index (BMI) of 22.5-25. We then used the number of cause-specific deaths to aggregate the BMI intervals of 2.5 that have been used in the studies to the WHO classification of BMI ranges,[127] i.e. BMI < 18.5: underweight; 18.5-24.9: normal range; 25-29.9: overweight; and 30-39.9: obesity.

A.2.4 Environmental analysis

For our environmental analysis, we used a food systems model that connects food consumption and production across regions.[128] The model distinguishes several steps along the food chain: primary production, trade in primary commodities, processing to oils, oil cakes and refined sugar, use of feed for animals, and trade in processed commodities and animals. It is parameterised with data from the International Model for Policy Analysis of Agricultural Commodities and Trade (IMPACT) [1] on current and future food production, processing factors, and feed requirements for 62 agricultural commodities and 159 countries. Projections of future food consumption and production were based on statistical association with changes in income and population, and were in line with other projections [3]. Below we summarise the main model equations. A full description of the IMPACT-related parameters is provided elsewhere [1].

Food systems model

Because our focus is on the environmental impacts of different diet scenarios, we did not account for non-food uses of agricultural commodities (e.g. by industry or as biofuels) in this study.

We first calculated the feed demand that supports the consumption of animal-based foods in the specific dietary scenarios. Because feed requirements differ by region, we first estimated where livestock is produced by accounting for trade flows ($QL_{c,r}^{trd} = QL_{c,r} - QL_{c,r}^{imp} + QL_{c,r}^{exp}$). For that purpose, we used import-to-demand fractions ($FI_{c,r} = \frac{QI_{c,r}}{QD_{c,r}^{cns+oth}}$) to calculate the percentage of livestock that is imported ($QL_{c,r}^{imp}$), and balanced imports with exports ($QL_{c,r}^{\mathrm{exp}}$) in line with projected imports and exports ($QI_{c,r}$, $QE_{c,r}$) by using the ratio of regional exports to all exports ($FE_{c,r} = \frac{QE_{c,r}}{\sum_r QE_{c,r}}$), a method that implicitly assumes that in each dietary scenario, current exporters stay exporters, and current importers stay importers. Feed demand ($QF_{c,r}$) is then calculated in relation to regional feed requirements ($FR_{c,r}$):

$$QL_{c,r}^{imp} = FI_{c,r} \cdot QL_{c,r}$$

$$QL_{c,r}^{\mathrm{exp}} = FE_{c,r} \cdot \sum_r QL_{c,r}^{imp}$$

$$QF_{c,r} = FR_{c,r} \cdot QL_{c,r}^{trd}$$

Next we calculate the intermediate demand for primary commodities that supports the consumption of processed goods (vegetable oils, oil meals, refined sugar) in the dietary scenarios. For that purpose, we first adjusted the mix of intermediate processed commodities for trade ($P_{c,r}^{trd} = QP_{c,r} - QP_{c,r}^{imp} + QP_{c,r}^{exp}$), and then used region-specific processing factors for oils and sugar ($PF_{c,r}$) to calculate the demand for primary commodities (oil crops, sugar crops):

$$QInt_{c,r} = PF_{c,r} \cdot QP_{c,r}^{trd}$$

Finally, we accounted for trade in those primary commodities that satisfy the demand for processing ($QInt_{c,r}^{trd} = QInt_{c,r} - QInt_{c,r}^{imp} + QInt_{c,r}^{exp}$), in feed that consists of primary commodities ($QF_{c,r}^{trd} = QF_{c,r} - QF_{c,r}^{imp} + QF_{c,r}^{exp}$), and in the primary commodities that are demanded in unprocessed form ($QD_{c,r}^{cns,trd} = QD_{c,r}^{cns} - QD_{c,r}^{cns,imp} + QD_{c,r}^{cns,exp}$). The production of primary commodities is then given by the sum of:

$$QS_{c,r} = QD_{c,r}^{cns,trd} + QF_{c,r}^{trd} + QInt_{c,r}^{trd} - QL_{c,r} - QP_{c,r}$$

Environmental footprints

To assess the environmental impacts of the dietary changes, we paired the production estimates of the diet scenarios with a set of country-specific environmental footprints related to GHG emissions, cropland use, freshwater use, and nitrogen and phosphorus application. Supplementary Table 10 provides an overview of the environmental footprints in the baseline. Future footprints take into account feasible changes in technologies and management.[128]

For GHG emissions, we focused on the non-CO_2 emissions of agriculture, in particular methane and nitrous oxide, in line with methodology followed by the International Panel on Climate Change. Data on GHG emissions were adopted from country-specific analyses of GHG emissions from crops,[129] and livestock.[130] Non-CO_2 emissions of fish and seafood were calculated based on feed requirements and feed-related emissions of aquaculture,[131] and on projections of the ratio between wild-caught and farmed fish production.[132,133] For future years, we incorporated the mitigation potential of bottom-up changes in management practices and technologies by using marginal abatement cost curves,[134] and the value of the social cost of carbon (SCC) in that year.[135] The mitigation options included changes in irrigation, cropping and fertilization that reduce methane and nitrous oxide emissions for rice and other crops, as well as changes in manure management, feed conversion and feed additives that reduce enteric fermentation in livestock.

Data on cropland and consumptive freshwater use from surface and groundwater (also termed blue water) were adopted from the IMPACT model for a range of different socio-economic pathways.[1] To derive commodity-specific footprints, we divided use data by data on primary production, and we calculated the footprints of processed goods (vegetable oils, refined sugar) by using country-specific conversion ratios,[1] and splitting coproducts (oils and oil meals) by economic value to avoid double counting. We used country-specific feed requirements for terrestrial animals [1] to derive the cropland and blue-water footprints for meat and dairy, and we used global feed requirements for aquaculture [131] and projections of the ratio between wild-caught and farmed fish production [132,133] to derive the cropland and blue-water footprints for fish and seafood. For future years, we included efficiency gains in agricultural yields, water management, and feed conversion that were based on IMPACT projections.[1] For water management, we relied on an integrated hydrological model within

IMPACT that operates at the level of watersheds and accounts for management changes that increase basin efficiency, storage capacity, and better utilization of rainwater.[1] For agricultural yields, the gains in land-use efficiency by 2050 matched estimates of yield-gap closures of about 75% between current yields and yields that are feasible in a given agro-climatic zone.[136]

Data on fertilizer application rates of nitrogen and phosphorous were adopted from the International Fertilizer Industry Association [137]. For future years, we included efficiency gains in nitrogen and phosphorus application from rebalancing of fertilizer application rates between over and under-applying regions in line with closing yield gaps.[136] In addition, we included improvements in nitrogen use efficiency of 15% by 2030 and 30% by 2050, in line with targets suggested by the Global Nitrogen Assessment,[138] and we included recycling rates of phosphorus of 25% by 2030 and 50% by 2050.[139]

For our uncertainty analysis, we incorporated different socio-economic pathways (SSPs) that influence food demand, including a middle-of-the-road development pathway (SSP2), a more optimistic pathway with higher income and lower population growth (SSP1), and a more pessimistic pathway with lower income and greater population growth (SSP3).[140–142] Supplementary Table 11 provides an overview of the different SSPs.

Supplementary Table 10. Environmental footprints of food commodities (per kg of product) (global averages). Footprints for animal products represent feed-related impacts, except for GHG emissions of livestock which also have a direct component. Footprints for fish and seafood represent feed-related impacts of aquaculture production weighted by total production volumes.

Food item	GHG intensity (kgCO2/kg)	Cropland use (m2/kg)	Freshwater use (m3/kg)	Nitrogen use (kgN/t)	Phosphorus use (kgP/t)
wheat	0.23	3.36	0.49	28.73	4.39
rice	1.18	3.51	1.07	36.64	5.20
maize	0.19	1.98	0.15	22.77	3.57
other grains	0.29	6.14	0.17	16.36	2.71
roots	0.07	0.69	0.04	3.63	0.71
legumes	0.23	11.02	0.95		
soybeans	0.12	3.95	0.14	2.75	5.88
nuts & seeds	0.71	6.39	0.43	14.27	2.11
vegetables	0.06	0.49	0.09	9.55	1.67
fruits (temperate)	0.08	1.18	0.33	12.73	1.91
fruits (tropical)	0.09	0.94	0.32	10.27	1.58
fruits (starchy)	0.11	0.85	0.12	6.26	1.07
sugar crops	0.02	0.15	0.11	2.03	0.35
oil crops	0.46	5.45	0.31	31.33	5.61
palm crop	0.38	0.63	0.00	4.57	0.73
sugar	0.19	1.67	1.22	22.34	3.84
palm oil	1.85	3.10	0.00	22.33	3.57
vegetable oil	0.67	10.31	0.47	42.73	11.47
beef	32.49	4.21	0.22	27.29	5.36
lamb	33.02	6.24	0.49	27.51	4.94
pork	2.92	6.08	0.35	51.52	8.87
poultry	1.41	6.59	0.40	50.20	9.02
eggs	1.58	6.86	0.44	51.22	8.81
milk	1.22	1.34	0.08	6.32	1.58
shellfish	0.07	0.36	0.03	3.35	0.81
fish (freshwater)	0.30	1.51	0.10	16.78	3.62
fish (demersal)	0.02	0.12	0.01	1.20	0.29
fish (pelagic)	0.00	0.00	0.00	0.00	0.00

Supplementary Table 11. Overview of income and population changes in the socio-economic development pathways. The pathways include a middle-of-the-road development pathway (shared socio-economic pathway 2, SSP2), a more optimistic pathway with higher income and lower population growth (SSP1), and a more pessimistic pathway with lower income and greater population growth (SSP3). Baseline conditions in 2010 are denoted by BMK(2010).

Region and parameter	Scenario (year)			
	BMK (2010)	SSP2 (2050)	SSP1 (2050)	SSP3 (2050)
East Asia and Pacific				
GDP	19,236	80,045	104,096	60,608
Population	2,184	2,261	2,173	2,351
GDP per capita	9	35	48	26
Europe				
GDP	14,628	27,780	30,571	21,342
Population	537	577	592	498
GDP per capita	27	48	52	43
Former Soviet Union (excl. Baltic States)				
GDP	2,855	8,984	10,603	7,551
Population	279	277	262	289
GDP per capita	10	32	40	26
Latin America and Caribbean				
GDP	5,834	19,164	22,838	15,894
Population	585	742	674	853
GDP per capita	10	26	34	19
Middle East and North Africa				
GDP	4,551	18,631	20,566	16,006
Population	457	715	646	808
GDP per capita	10	26	32	20
North America				
GDP	14,290	29,933	33,691	24,753
Population	344	450	460	372
GDP per capita	41	67	73	67
South Asia				
GDP	4,461	32,939	44,250	22,756
Population	1,630	2,373	2,108	2,720
GDP per capita	3	14	21	8
Sub-Saharan Africa				
GDP	1,705	13,962	19,690	9,665
Population	863	1,793	1,564	2,084
GDP per capita	2	8	13	5
World				
GDP	67,559	231,439	286,305	178,575
Population	6,879	9,187	8,479	9,975
GDP per capita	10	25	34	18

Source: Calculated from IMPACT 3.1 with population and GDP growth rates from IIASA and OECD

Note: GDP and GDP per capita are in purchasing power parity (ppp)

A.3 Supplementary results

Supplementary Table 12. Mean, low, and high values of globally averaged nutrient levels in 2010.

Nutrient	Stats	Diet scenarios												
		BMK	FLX	PSC	VEG	VGN	ani-25	ani-50	ani-75	ani-100	kcal-25	kcal-50	kcal-75	kcal-100
calories	mean	2145.7	2083.9	2083.8	2084.4	2084.4	2156.8	2157.0	2157.2	2157.3	2138.4	2120.2	2102.0	2083.8
	low	2064.5	1980.6	1979.0	1998.0	2001.5	2074.7	2078.5	2082.3	2086.2	2053.9	2037.0	2020.0	2003.1
	high	2286.1	2203.4	2204.6	2163.8	2159.2	2286.5	2268.7	2250.9	2233.1	2283.6	2262.9	2242.2	2221.5
protein	mean	68.4	70.6	72.5	65.0	64.7	67.9	66.6	65.3	64.1	68.5	67.8	67.2	66.5
	low	61.6	62.7	64.0	59.0	58.4	61.1	60.3	59.4	58.5	61.4	60.9	60.3	59.8
	high	75.3	78.7	82.2	71.5	71.3	75.2	73.8	72.4	71.0	75.8	75.0	74.2	73.4
carbohydrates	mean	324.4	273.8	278.1	288.7	303.7	341.1	356.1	371.1	386.1	323.9	321.8	319.6	317.5
	low	313.1	260.9	264.2	274.6	289.8	328.2	342.3	356.4	370.5	312.1	310.1	308.2	306.2
	high	344.6	294.2	300.2	308.6	319.5	360.9	374.5	388.1	401.7	344.9	342.4	340.0	337.6
fat	mean	68.9	81.8	78.1	77.3	71.3	62.7	56.4	50.1	43.8	68.1	67.2	66.4	65.5
	low	59.3	74.0	72.1	72.8	67.5	54.7	50.2	45.7	41.1	58.6	57.9	57.2	56.5
	high	79.1	90.6	87.2	81.8	76.2	71.7	64.0	56.3	48.6	78.4	77.3	76.2	75.2
saturatedFA	mean	22.5	19.7	17.5	17.2	13.4	19.3	16.0	12.7	9.5	22.3	22.0	21.7	21.4
	low	19.1	17.3	16.1	16.3	12.9	16.6	14.2	11.7	9.2	18.9	18.7	18.5	18.3
	high	30.7	32.5	30.1	28.6	24.7	27.0	23.1	19.3	15.5	30.3	29.9	29.5	29.1
monounsatFA	mean	26.7	31.4	28.1	27.7	26.1	23.7	20.7	17.7	14.6	26.4	26.1	25.7	25.4
	low	20.9	25.1	23.8	24.0	22.6	18.9	16.8	14.8	12.8	20.7	20.4	20.2	19.9
	high	31.1	36.8	34.0	31.4	29.7	27.5	23.8	20.2	16.6	30.7	30.3	29.9	29.4
polyunsatFA	mean	16.7	27.7	27.2	27.4	27.6	16.7	16.8	16.8	16.8	16.5	16.3	16.1	15.9
	low	11.9	19.4	19.0	19.6	19.8	12.1	12.3	12.5	12.6	11.8	11.7	11.6	11.4
	high	18.9	31.3	31.2	30.3	30.6	18.9	18.7	18.6	18.5	18.7	18.5	18.2	18.0
vitaminC	mean	86.9	148.3	162.5	170.9	195.7	124.1	146.8	169.5	192.3	100.3	99.3	98.2	97.2
	low	64.3	102.4	111.3	117.7	133.2	87.6	102.4	117.1	131.9	72.1	71.3	70.6	69.9
	high	189.5	366.8	417.8	388.8	447.5	271.3	321.5	371.6	421.8	218.9	216.6	214.4	212.1
vitaminA	mean	482.1	626.6	679.3	694.2	702.8	627.4	680.3	733.2	786.2	568.1	561.7	555.4	549.0
	low	270.8	357.1	385.3	400.5	385.2	345.5	368.5	391.6	414.6	318.6	314.8	310.9	307.1
	high	943.7	1526.7	1750.7	1621.8	1750.1	1341.9	1496.7	1651.6	1806.4	1172.8	1158.5	1144.2	1129.8
folate	mean	280.3	553.2	576.6	643.5	733.1	410.1	504.1	598.1	692.2	313.2	310.3	307.4	304.4
	low	248.7	480.2	494.0	560.7	636.3	350.4	430.6	510.8	591.1	267.9	265.6	263.4	261.1
	high	330.4	668.8	704.4	776.5	888.7	501.7	619.1	736.4	853.8	380.8	377.1	373.5	369.9
calcium	mean	555.7	621.0	660.4	629.8	489.2	546.3	517.5	488.7	459.9	571.3	567.5	563.7	559.9
	low	397.5	438.3	452.4	466.3	382.2	393.1	379.6	366.0	352.5	404.3	402.0	399.7	397.4
	high	802.6	1111.4	1285.9	1066.0	972.5	897.6	899.1	900.5	902.0	889.3	882.4	875.6	868.7
iron	mean	16.4	18.8	19.3	19.5	21.1	18.1	19.3	20.5	21.6	16.9	16.8	16.7	16.6
	low	12.6	14.2	14.4	15.0	16.2	13.8	14.7	15.5	16.4	12.8	12.8	12.7	12.6
	high	27.0	32.3	35.9	31.8	35.1	30.5	32.5	34.4	36.4	28.3	28.1	27.9	27.7
zinc	mean	10.8	10.4	10.4	10.2	10.3	10.8	10.6	10.5	10.4	10.8	10.7	10.6	10.5
	low	9.5	9.1	8.9	9.1	9.1	9.5	9.4	9.3	9.2	9.5	9.4	9.3	9.3
	high	19.1	16.8	20.0	12.6	12.6	17.7	16.1	14.5	13.0	19.0	18.8	18.5	18.2
potassium	mean	2506.1	3383.0	3555.0	3633.9	3952.4	2951.4	3282.7	3614.1	3945.4	2595.4	2570.8	2546.3	2521.7
	low	2227.0	3022.5	3144.4	3275.9	3570.4	2632.2	2939.6	3246.9	3554.3	2305.0	2285.0	2265.1	2245.2
	high	3109.5	4044.3	4273.2	4318.1	4604.7	3641.7	3986.3	4331.0	4675.7	3262.8	3228.6	3194.3	3160.1
fiber	mean	26.0	35.5	36.6	39.9	44.6	31.5	36.1	40.7	45.4	26.6	26.3	26.0	25.8
	low	23.9	31.4	32.1	35.0	38.9	28.1	31.9	35.8	39.7	23.9	23.7	23.5	23.2
	high	30.7	41.8	43.2	47.2	52.9	37.8	43.6	49.3	55.1	31.7	31.4	31.1	30.8
copper	mean	1.6	2.3	2.3	2.5	2.7	1.8	2.1	2.3	2.5	1.6	1.6	1.6	1.6
	low	1.4	2.0	2.0	2.2	2.4	1.6	1.8	2.0	2.2	1.4	1.4	1.4	1.4
	high	2.4	3.6	4.0	3.8	4.2	3.0	3.4	3.7	4.1	2.6	2.6	2.6	2.6
phosphorus	mean	1311.7	1379.0	1428.8	1366.4	1337.1	1333.6	1347.1	1360.7	1374.2	1310.1	1300.2	1290.3	1280.4
	low	1124.2	1179.7	1201.8	1188.2	1187.6	1151.2	1171.7	1192.1	1212.5	1123.3	1115.7	1108.2	1100.6
	high	1610.1	1767.5	1915.8	1683.9	1670.2	1662.5	1682.9	1703.2	1723.6	1627.5	1612.8	1598.2	1583.5
thiamin	mean	1.3	1.5	1.5	1.5	1.6	1.4	1.5	1.6	1.6	1.3	1.3	1.3	1.3
	low	1.1	1.2	1.2	1.3	1.4	1.1	1.2	1.3	1.3	1.1	1.1	1.1	1.0
	high	2.1	2.2	2.2	2.2	2.2	2.2	2.2	2.2	2.3	2.1	2.1	2.0	2.0
riboflavin	mean	0.9	0.9	1.0	0.9	0.9	0.9	0.9	0.9	0.9	0.9	0.9	0.9	0.9
	low	0.7	0.7	0.7	0.7	0.7	0.7	0.7	0.7	0.7	0.7	0.7	0.7	0.7
	high	1.8	1.9	2.1	1.8	1.6	1.8	1.8	1.7	1.7	1.8	1.8	1.8	1.8
niacin	mean	18.7	17.5	17.4	16.0	16.8	18.6	18.4	18.1	17.9	18.7	18.5	18.3	18.1
	low	14.7	14.0	13.5	13.3	14.0	14.7	14.6	14.5	14.3	14.8	14.6	14.5	14.4
	high	28.2	26.0	27.8	23.0	25.3	28.5	28.5	28.5	28.5	28.0	27.7	27.3	26.9
vitaminB6	mean	6.1	6.1	6.2	6.1	2.3	5.2	4.3	3.3	2.4	6.2	6.2	6.3	6.4
	low	5.9	5.9	5.9	5.9	2.1	5.0	4.1	3.1	2.2	6.0	6.0	6.1	6.2
	high	6.5	6.7	6.8	6.5	2.7	5.7	4.7	3.8	2.9	6.7	6.7	6.8	6.9
magnesium	mean	436.3	527.1	543.3	561.1	596.1	489.3	528.3	567.3	606.3	447.8	445.3	442.9	440.4
	low	404.4	479.3	489.6	510.2	544.5	446.8	481.5	516.3	551.0	410.0	407.9	405.9	403.8
	high	493.3	613.6	649.7	640.0	683.9	557.5	601.5	645.5	689.4	510.6	507.8	504.9	502.1
pantothenate	mean	5.7	5.4	5.4	5.3	4.9	6.4	6.1	5.8	5.5	6.7	6.7	6.7	6.6
	low	5.7	5.4	5.4	5.3	4.9	6.4	6.1	5.8	5.5	6.7	6.7	6.7	6.6
	high	5.7	5.4	5.4	5.3	4.9	6.4	6.1	5.8	5.5	6.7	6.7	6.7	6.6
vitaminB12	mean	3.0	2.4	3.7	0.8	0.0	2.8	1.8	0.9	0.0	3.7	3.6	3.6	3.6
	low	3.0	2.4	3.7	0.8	0.0	2.8	1.8	0.9	0.0	3.7	3.6	3.6	3.6
	high	3.0	2.4	3.7	0.8	0.0	2.8	1.8	0.9	0.0	3.7	3.6	3.6	3.6

Supplementary Table 13. Percentage differences to nutrient recommendations. Red indicates nutrient levels below recommended values for minimal intake, and black levels indicates levels above recommended values for maximum intake. Blanks indicate that recommendations are met.

Region	Nutrient	Diet scenarios												
		BMK	FLX	PSC	VEG	VGN	ani-25	ani-50	ani-75	ani-100	kcal-25	kcal-50	kcal-75	kcal-100
Global	vitaminA	-11												
	folate	-23									-14	-15	-16	-16
	calcium					-6		-1	-6	-12				
	iron	-6									-3	-4	-5	-5
	potassium	-23					-9				-20	-21	-22	-22
	fiber	-11									-9	-10	-11	-12
	riboflavin	-18	-15	-13	-15	-21	-17	-18	-19	-20	-17	-18	-19	-20
	vitaminB12				-66	-100		-16	-58	-100				
HIC	saturatedFA	65					36	7			62	59	56	52
	vitaminA	-3												
	folate	-30									-23	-24	-26	-27
	calcium					-13				-1				
	iron	-26					-12	-0			-25	-26	-27	-28
	potassium	-25					-4				-24	-26	-27	-28
	fiber	-14									-13	-15	-16	-18
	riboflavin					-2								
	pantothenate					-4				-1				
	vitaminB12				-52	-100			-38	-100				
UMC	saturatedFA	14									11	9	6	3
	folate	-16									-11	-13	-16	-18
	calcium					-7				-7				
	iron	-12					-1				-12	-14	-17	-19
	potassium	-7									-7	-9	-12	-14
	pantothenate					-7				-3				
	vitaminB12				-48	-100		-13	-57	-100				
LMC	polyunsatFA													-1
	vitaminA	-15												
	folate	-20									-11	-12	-13	-14
	calcium	-5				-3	-4	-6	-8	-10	-2	-2	-3	-3
	iron	-2										-1	-2	-3
	potassium	-23	-1				-11	-2			-21	-22	-23	-24
	fiber	-14									-13	-14	-15	-16
	riboflavin	-26	-26	-24	-26	-33	-26	-27	-28	-29	-26	-27	-27	-28
	vitaminB12				-69	-100	-10	-40	-70	-100				
LIC	protein	-2								-1				
	polyunsatFA	-30					-29	-29	-30	-30	-27	-26	-25	-24
	vitaminA	-38					-23	-19	-15	-12	-25	-23	-22	-20
	folate	-32					-7				-16	-14	-12	-10
	calcium	-33				-7	-29	-31	-32	-34	-25	-23	-21	-19
	potassium	-32					-19	-15	-11	-7	-21	-19	-18	-16
	fiber	-17					-3				-7	-5	-3	-0
	riboflavin	-41	-15	-14	-14	-19	-36	-37	-38	-39	-34	-33	-31	-30
	vitaminB12	-41	-32	-14	-82	-100			-36	-100				

Supplementary Table 14. Global number of averted deaths and averted premature deaths in 2030 (in thousands) by risk factor. Values are reported as mean and lower and upper value of 95% confidence intervals.

Risk factor	Stats	FLX	PSC	VEG	VGN	ani-25	ani-50	ani-75	ani-100	kcal-25	kcal-50	kcal-75	kcal-100
Total deaths averted (thousands)													
all risks	mean	11,155	11,905	11,297	12,766	2,447	4,146	5,583	6,968	1,315	2,683	4,074	5,622
	low	10,379	10,983	10,284	11,644	2,231	3,746	5,006	6,220	1,160	2,396	3,676	5,125
	high	11,931	12,826	12,309	13,888	2,663	4,546	6,159	7,717	1,471	2,971	4,473	6,120
vegetables	mean	1,010	1,263	1,409	2,118	1,096	1,927	2,692	3,553	-168	-362	-560	-599
	low	961	1,209	1,350	2,037	1,058	1,862	2,602	3,436	-163	-351	-543	-578
	high	1,058	1,316	1,468	2,198	1,135	1,993	2,781	3,670	-173	-373	-578	-620
fruits	mean	1,032	1,196	1,376	2,038	784	1,236	1,589	1,893	-126	-253	-399	-413
	low	951	1,106	1,276	1,895	727	1,150	1,481	1,768	-116	-232	-367	-383
	high	1,112	1,285	1,476	2,181	842	1,323	1,697	2,017	-136	-273	-431	-444
nuts & seeds	mean	1,229	1,229	1,229	1,229	0	0	0	0	-67	-41	7	-75
	low	1,160	1,160	1,160	1,160	0	0	0	0	-64	-36	8	-69
	high	1,299	1,299	1,299	1,299	0	0	0	0	-69	-46	6	-80
legumes	mean	1,185	1,185	1,611	2,163	612	1,169	1,684	2,159	-11	-31	-51	-71
	low	956	956	1,300	1,745	496	947	1,364	1,750	-9	-25	-41	-57
	high	1,414	1,414	1,922	2,580	728	1,391	2,003	2,569	-13	-37	-60	-84
fish	mean	351	878	-1,047	-1,047	-226	-482	-758	-1,047	-28	-71	-115	-159
	low	218	549	-670	-670	-144	-309	-485	-670	-18	-46	-74	-102
	high	485	1,207	-1,423	-1,423	-307	-656	-1,031	-1,423	-38	-97	-156	-216
red meat	mean	824	964	964	964	251	496	733	964	45	91	137	183
	low	781	913	913	913	238	470	695	913	43	87	130	174
	high	866	1,015	1,015	1,015	264	522	772	1,015	47	96	145	193
underweight	mean	1,937	1,937	1,937	1,937					484	969	1,453	1,937
	low	1,841	1,841	1,841	1,841					456	916	1,379	1,841
	high	2,034	2,034	2,034	2,034					513	1,021	1,527	2,034
overweight	mean	868	868	868	868					217	434	651	868
	low	802	802	802	802					200	400	601	802
	high	935	935	935	935					235	468	702	935
obese	mean	3,864	3,864	3,864	3,864					966	1,932	2,898	3,864
	low	3,519	3,519	3,519	3,519					867	1,744	2,631	3,519
	high	4,208	4,208	4,208	4,208					1,065	2,120	3,164	4,208
Premature deaths averted (thousands)													
all risks	mean	4,355	4,655	4,518	5,092	902	1,554	2,114	2,645	535	1,077	1,624	1,989
	low	4,012	4,250	4,103	4,632	822	1,407	1,903	2,371	466	950	1,450	2,426
	high	4,698	5,061	4,933	5,552	981	1,700	2,325	2,919	605	1,204	1,799	-262
vegetables	mean	463	595	677	1,030	464	836	1,171	1,531	-65	-144	-225	-252
	low	438	566	646	986	445	803	1,126	1,473	-63	-139	-217	-272
	high	488	624	709	1,074	483	869	1,216	1,589	-67	-149	-233	-138
fruits	mean	395	475	546	804	283	464	626	766	-38	-80	-127	-126
	low	361	436	502	742	260	429	580	712	-35	-73	-115	-149
	high	430	514	589	866	306	499	672	819	-41	-87	-138	-40
nuts & seeds	mean	592	592	592	592	0	0	0	0	-21	-14	-5	-37
	low	552	552	552	552	0	0	0	0	-20	-12	-4	-43
	high	632	632	632	632	0	0	0	0	-23	-16	-5	-19
legumes	mean	364	364	491	661	169	320	461	591	-1	-7	-13	-14
	low	276	276	374	502	128	244	351	451	-1	-5	-10	-23
	high	451	451	609	819	209	397	571	732	-2	-9	-16	-40
fish	mean	135	308	-279	-279	-61	-129	-203	-279	-7	-18	-29	-22
	low	73	167	-154	-154	-33	-71	-111	-154	-4	-10	-16	-58
	high	197	450	-405	-405	-88	-187	-294	-405	-11	-26	-42	57
red meat	mean	249	299	299	299	78	154	228	299	14	28	42	52
	low	230	276	276	276	72	142	210	276	13	26	39	61
	high	268	322	322	322	84	166	245	322	15	30	46	920
underweight	mean	920	920	920	920					230	460	690	866
	low	866	866	866	866					214	431	649	974
	high	974	974	974	974					246	489	731	349
overweight	mean	349	349	349	349					87	174	261	318
	low	318	318	318	318					79	158	238	380
	high	380	380	380	380					95	190	285	1,348
obese	mean	1,348	1,348	1,348	1,348					337	674	1,011	1,206
	low	1,206	1,206	1,206	1,206					296	596	901	1,491
	high	1,491	1,491	1,491	1,491					378	752	1,122	

Supplementary Table 15. Global and regional health in mortality (upper panel), premature mortality (middle panel), and years of life lost (lower panel). Regions include high-income countries (HIC), upper middle-income countries (UMC), lower middle-income countries (LMC), low-income countries (LIC), and an aggregate of all countries (Global). Values are reported as mean and lower and upper value of 95% confidence intervals.

Region	Stats	FLX	PSC	VEG	VGN	ani-25	ani-50	ani-75	ani-100	kcal-25	kcal-50	kcal-75	kcal-100
Total deaths averted (thousands)													
Global	mean	11,155	11,905	11,297	12,766	2,447	4,146	5,583	6,968	1,315	2,683	4,074	5,622
	low	10,379	10,983	10,284	11,644	2,231	3,746	5,006	6,220	1,160	2,396	3,676	5,125
	high	11,931	12,826	12,309	13,888	2,663	4,546	6,159	7,717	1,471	2,971	4,473	6,120
HIC	mean	1,931	2,017	1,876	1,991	394	605	769	983	286	586	866	1,210
	low	1,810	1,873	1,725	1,827	360	543	679	867	261	539	802	1,131
	high	2,051	2,161	2,027	2,155	428	668	858	1,100	312	633	930	1,290
UMC	mean	2,038	2,172	2,033	2,216	407	684	891	1,103	269	594	838	1,116
	low	1,868	1,968	1,835	1,997	367	611	789	971	231	526	745	1,000
	high	2,207	2,376	2,231	2,434	446	757	994	1,234	306	663	932	1,232
LMC	mean	5,834	6,321	6,008	7,146	1,493	2,602	3,606	4,501	530	1,068	1,763	2,490
	low	5,441	5,853	5,442	6,509	1,364	2,359	3,251	4,039	458	930	1,567	2,244
	high	6,226	6,789	6,574	7,784	1,623	2,846	3,961	4,964	603	1,207	1,959	2,736
LIC	mean	1,378	1,420	1,400	1,449	151	257	333	405	230	434	625	825
	low	1,285	1,315	1,299	1,343	137	232	300	362	209	400	580	768
	high	1,471	1,526	1,501	1,555	165	281	367	449	250	468	669	881
Premature deaths averted (thousands)													
Global	mean	4,355	4,655	4,518	5,092	902	1,554	2,114	2,645	535	1,077	1,624	2,208
	low	4,012	4,250	4,103	4,632	822	1,407	1,903	2,371	466	950	1,450	1,989
	high	4,698	5,061	4,933	5,552	981	1,700	2,325	2,919	605	1,204	1,799	2,426
HIC	mean	442	466	447	486	109	178	235	302	61	124	183	255
	low	409	427	407	443	101	162	212	272	54	111	166	234
	high	475	504	487	529	118	194	258	332	68	137	201	277
UMC	mean	749	794	763	832	153	261	345	426	101	220	314	418
	low	677	709	681	743	138	234	306	377	85	190	273	367
	high	821	879	845	922	168	289	383	476	118	250	355	468
LMC	mean	2,451	2,663	2,578	3,025	564	990	1,376	1,725	250	501	799	1,100
	low	2,266	2,440	2,340	2,755	516	898	1,242	1,550	216	437	709	987
	high	2,636	2,887	2,816	3,295	613	1,082	1,510	1,900	284	565	888	1,213
LIC	mean	714	733	731	754	74	126	164	200	122	229	330	436
	low	662	675	676	697	67	114	148	179	110	210	305	403
	high	766	791	786	812	81	138	181	222	133	248	356	468
Years of life saved (thousands)													
Global	mean	265,845	283,191	272,016	305,545	54,361	92,869	125,732	157,101	33,335	67,470	101,875	139,241
	low	246,174	259,970	247,433	278,375	49,487	83,852	112,744	140,253	29,315	60,116	91,726	126,534
	high	285,516	306,412	296,600	332,715	59,235	101,886	138,721	173,948	37,355	74,824	112,024	151,948
HIC	mean	36,119	37,851	35,627	38,154	7,870	12,430	16,106	20,614	5,265	10,764	15,910	22,195
	low	33,663	34,941	32,588	34,863	7,193	11,186	14,328	18,302	4,736	9,803	14,600	20,566
	high	38,575	40,761	38,666	41,446	8,547	13,674	17,885	22,926	5,794	11,726	17,220	23,824
UMC	mean	46,389	49,314	46,736	50,947	9,213	15,620	20,500	25,371	6,270	13,705	19,489	25,945
	low	42,282	44,425	41,989	45,737	8,299	13,943	18,134	22,340	5,353	12,023	17,200	23,107
	high	50,496	54,203	51,483	56,158	10,126	17,297	22,866	28,403	7,187	15,387	21,779	28,783
LMC	mean	142,382	154,007	147,834	173,686	33,207	58,088	80,624	100,847	14,479	29,119	46,776	65,001
	low	132,209	141,850	134,061	158,179	30,288	52,586	72,588	90,385	12,572	25,531	41,725	58,644
	high	152,554	166,165	161,606	189,194	36,127	63,591	88,660	111,310	16,386	32,707	51,827	71,357
LIC	mean	41,370	42,451	42,152	43,447	4,043	6,833	8,920	10,864	7,287	13,818	20,009	26,418
	low	38,462	39,201	39,054	40,202	3,649	6,178	8,012	9,691	6,625	12,704	18,519	24,541
	high	44,277	45,700	45,250	46,693	4,436	7,489	9,827	12,037	7,950	14,932	21,499	28,296

Supplementary Figure 1. Global distribution of averted deaths by disease (%) by diet scenario.

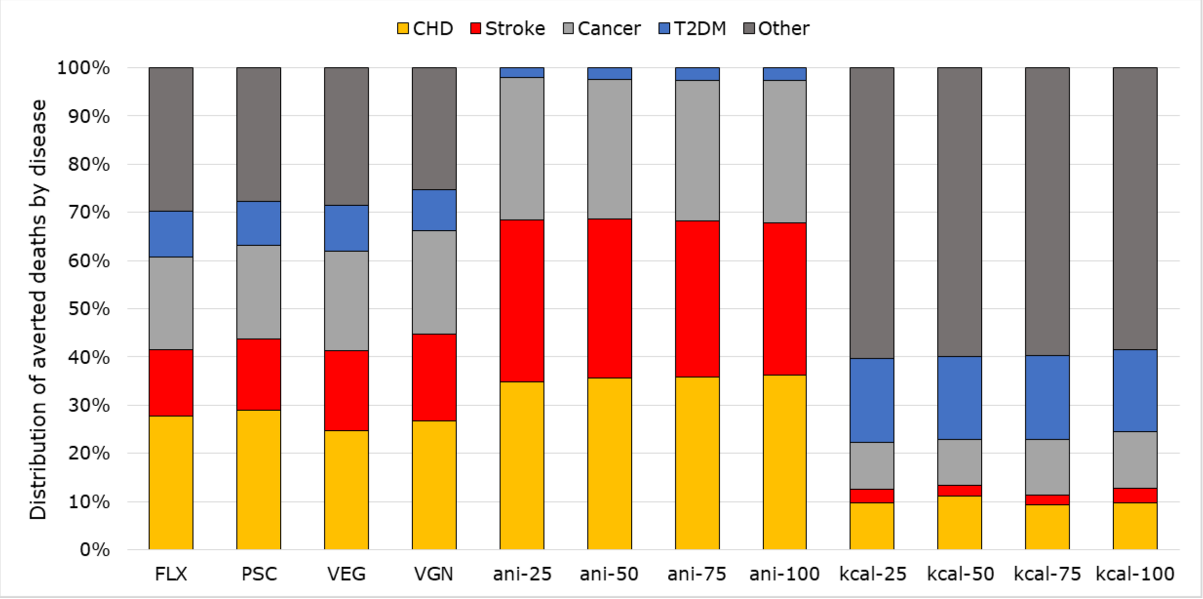

Supplementary Table 16. Global environmental impacts in 2030 by socio-economic development scenario (upper panel) and region (lower panel, for socio-economic scenario SSP2).

Environmental domain	Socio-economic scenario	Diet scenario												
		BMK	FLX	PSC	VEG	VGN	ani-25	ani-50	ani-75	ani-100	kcal-25	kcal-50	kcal-75	kcal-100
GHG emissions (MtCO2-eq)	SSP2	7,323	3,353	1,799	1,807	963	5,828	4,269	2,710	1,151	7,170	6,953	6,735	6,518
	SSP1	7,402	3,307	1,774	1,781	937	5,889	4,307	2,725	1,143	7,222	6,974	6,726	6,477
	SSP3	7,221	3,380	1,812	1,819	982	5,753	4,222	2,690	1,159	7,089	6,894	6,700	6,505
Cropland use (M km2)	SSP2	10,251	9,382	9,113	9,252	9,166	10,297	10,133	9,968	9,803	10,163	9,864	9,564	9,265
	SSP1	10,233	9,192	8,921	9,054	8,961	10,277	10,106	9,936	9,765	10,110	9,771	9,433	9,095
	SSP3	10,269	9,535	9,269	9,413	9,334	10,321	10,164	10,007	9,851	10,205	9,932	9,659	9,386
Freshwater use (km3)	SSP2	1,506	1,343	1,354	1,388	1,480	1,568	1,629	1,689	1,749	1,476	1,444	1,412	1,379
	SSP1	1,500	1,314	1,324	1,358	1,447	1,562	1,622	1,682	1,742	1,465	1,428	1,390	1,353
	SSP3	1,511	1,368	1,379	1,415	1,510	1,574	1,635	1,696	1,757	1,484	1,456	1,427	1,398
Nitrogen application (GgN)	SSP2	76,626	58,860	58,284	57,399	57,180	76,181	75,471	74,761	74,051	74,242	71,593	68,944	66,295
	SSP1	76,573	57,872	57,270	56,380	56,303	76,137	75,424	74,712	74,000	73,918	70,987	68,056	65,124
	SSP3	76,689	59,559	59,012	58,119	57,811	76,260	75,571	74,882	74,193	74,484	72,019	69,554	67,088
Phosphorus application (GgP)	SSP2	11,985	9,803	9,739	9,577	9,436	11,906	11,787	11,668	11,549	11,612	11,199	10,786	10,373
	SSP1	11,992	9,637	9,571	9,408	9,294	11,917	11,801	11,685	11,569	11,576	11,119	10,661	10,204
	SSP3	11,976	9,925	9,865	9,700	9,543	11,898	11,781	11,664	11,547	11,631	11,247	10,863	10,479

Environmental domain	Region	Diet scenario												
		BMK	FLX	PSC	VEG	VGN	ani-25	ani-50	ani-75	ani-100	kcal-25	kcal-50	kcal-75	kcal-100
GHG emissions (MtCO2-eq)	Global	7,323	3,353	1,799	1,807	963	5,828	4,269	2,710	1,151	7,170	6,953	6,735	6,518
	HIC	1,156	302	209	210	130	904	652	400	148	1,130	1,104	1,078	1,052
	UMC	1,411	384	216	218	99	1,086	761	436	111	1,354	1,296	1,238	1,180
	LMC	3,782	2,070	1,088	1,089	604	3,023	2,265	1,506	748	3,648	3,515	3,381	3,247
	LIC	992	609	296	299	135	830	603	376	150	1,056	1,055	1,054	1,054
Cropland use (M km2)	Global	10,251	9,382	9,113	9,252	9,166	10,297	10,133	9,968	9,803	10,163	9,864	9,564	9,265
	HIC	1,636	1,188	1,109	1,130	1,040	1,517	1,398	1,278	1,159	1,600	1,564	1,528	1,492
	UMC	1,563	1,233	1,158	1,198	1,182	1,517	1,471	1,426	1,380	1,503	1,444	1,384	1,325
	LMC	5,444	5,143	5,056	5,056	5,061	5,425	5,407	5,389	5,371	5,257	5,071	4,884	4,697
	LIC	1,695	1,883	1,845	1,922	1,931	1,917	1,928	1,940	1,951	1,885	1,865	1,844	1,824
Freshwater use (km3)	Global	1,506	1,343	1,354	1,388	1,480	1,568	1,629	1,689	1,749	1,476	1,444	1,412	1,379
	HIC	140	123	124	130	141	152	163	175	186	137	134	130	127
	UMC	133	111	113	118	130	143	152	162	171	128	123	117	112
	LMC	1,080	915	920	937	992	1,109	1,139	1,169	1,199	1,056	1,032	1,008	984
	LIC	154	196	199	206	222	167	177	187	197	157	157	158	158
Nitrogen application (GgN)	Global	76,626	58,860	58,284	57,399	57,180	76,181	75,471	74,761	74,051	74,242	71,593	68,944	66,295
	HIC	12,096	7,769	7,141	7,137	6,569	10,949	9,802	8,655	7,508	11,835	11,575	11,314	11,053
	UMC	8,074	5,699	5,321	5,304	5,121	7,638	7,201	6,765	6,329	7,757	7,440	7,123	6,806
	LMC	50,998	40,075	40,558	39,675	40,284	51,897	52,796	53,695	54,594	49,001	47,005	45,008	43,012
	LIC	6,080	5,797	5,717	5,724	5,623	6,292	6,238	6,184	6,131	6,244	6,144	6,043	5,942
Phosphorus application (GgP)	Global	11,985	9,803	9,739	9,577	9,436	11,906	11,787	11,668	11,549	11,612	11,199	10,786	10,373
	HIC	1,901	1,272	1,190	1,197	1,088	1,735	1,570	1,405	1,240	1,864	1,827	1,790	1,752
	UMC	1,609	1,205	1,142	1,135	1,061	1,511	1,413	1,314	1,216	1,539	1,468	1,398	1,327
	LMC	7,692	6,442	6,527	6,364	6,413	7,835	7,977	8,120	8,263	7,398	7,103	6,809	6,515
	LIC	867	948	941	937	925	904	900	896	892	893	878	864	849

Supplementary Table 17. Changes in environmental impacts by food group for three scenarios: ani-100 (upper panel), kcal-100 (middle panel), and FLX (lower panel). Results are shown for high-income countries (HIC), and low-income countries (LIC), and globally (Global).

Food group	GHG emissions (MtCO2-eq)			Cropland use (M km2)			Freshwater use (km3)			Nitrogen application (GgN)			Phosphorus application (GgP)		
	Global	HIC	LIC	Global	HIC	LIC	Global	HIC	LIC	Global	HIC	LIC	Global	HIC	LIC
total	-6172	-1008	-842	-448	-477	257	243	46	43	-2574	-4589	51	-436	-661	25
wheat	0		0	4	0	4	0		0	26	0	26	3	0	3
rice	0	0	0	-1		-1	0	0	0	-3		-3	0		0
maize	0		0	-1		-1	0		0	-2		-2	0		0
other grains	0		0	24		24	0		0	16		16	2		2
roots	2		2	13		13	0		0	11		11	1		1
legumes	45	14	5	1160	210	224	142	30	16						
soybeans	8	1	0	309	27	16	12	1	1	203	14	31	496	42	21
nuts & seeds	0		0	3		3	0		0						
vegetables	97	19	5	569	76	75	109	11	15	12007	984	624	1924	199	100
fruits (temperate)	23	9	1	312	85	69	87	24	18	2818	660	125	370	95	17
fruits (tropical)	33	5	1	299	50	33	98	22	8	3385	512	151	467	75	23
fruits (starchy)	2		2	33		33	1		1	84		84	12		12
sugar	0		0	0		0	0	0	0	-1		-1	0		0
beef	-3765	-586	-484	-308	-100	-23	-17	-5	-2	-2027	-858	-87	-376	-132	-15
lamb	-859	-105	-161	-119	-19	-13	-10	-1	-1	-560	-102	-37	-90	-15	-5
pork	-387	-118	-25	-635	-213	-41	-36	-10	-3	-4975	-1786	-241	-771	-244	-33
poultry	-185	-44	-12	-714	-206	-54	-46	-9	-2	-5381	-1742	-242	-882	-261	-37
eggs	-113	-23	-6	-406	-102	-20	-28	-5	-1	-2997	-903	-97	-463	-124	-13
milk	-1065	-178	-171	-882	-275	-71	-62	-12	-5	-4360	-1322	-250	-970	-287	-37
shellfish	-1	0	0	-13	-2	-1	-1	0	0	-90	-10	-6	-19	-2	-1
fish (freshwater)	-7	0	-1	-90	-4	-13	-7	0	-1	-685	-24	-49	-133	-5	-11
fish (demersal)	-1	0	0	-6	-2	0	0	0	0	-42	-12	0	-9	-3	0

Food group	GHG emissions (MtCO2-eq)			Cropland use (M km2)			Freshwater use (km3)			Nitrogen application (GgN)			Phosphorus application (GgP)		
	Global	HIC	LIC	Global	HIC	LIC	Global	HIC	LIC	Global	HIC	LIC	Global	HIC	LIC
total	-805	-104	62	-987	-144	129	-126	-14	4	-10331	-1043	-137	-1612	-148	-18
wheat	-12	-2	0	-150	-25	14	-13	-2	2	-1424	-180	66	-210	-20	8
rice	-62	-1	-6	-151	-2	-28	-40	-1	-3	-1641	-20	-229	-180	2	-30
maize	-3	0	0	-31	-1	-1	-1	0	1	-333	-17	-16	-41	-2	-2
other grains	-1	0	0	-20	-2	21	0	0	1	-77	-10	14	-12	-1	2
roots	-2	-1	1	-31	-2	5	-2	0	0	-316	-18	0	-50	-4	0
legumes	0	0	0	-4	-2	30	-2	0	1						
soybeans	0	0	0	-11	0	2	0	0	0	-8	0	0	-20	0	1
nuts & seeds	-3	0	0	-12	-1	1	-1	0	0	-7	-3	-1	-1	0	0
vegetables	-13	-1	0	-65	-3	4	-11	-1	1	-1918	-43	-1	-285	-6	0
fruits (temperate)	-2	0	0	-1	-4	23	-6	-1	1	-386	-27	6	-48	-4	1
fruits (tropical)	-5	0	0	-28	-2	5	-5	-2	0	-449	-23	2	-58	-3	0
fruits (starchy)	-1	0	1	19	0	26	-1	0	1	-36	-4	62	-5	-1	9
sugar	-4	-1	0	-29	-4	-1	-13	-1	0	-352	-34	-13	-55	-6	-2
palm oil	-8	0	0	-13	0	-1	0	0	0	-85	-4	-4	-12	0	-1
vegetable oil	-4	-1	0	-73	-15	-5	-3	-1	0	-249	-66	-5	-75	-11	-2
beef	-425	-54	34	-21	-9	20	-2	0	0	-269	-82	19	-55	-12	3
lamb	-84	-7	8	-15	-2	1	-1	0	0	-73	-11	1	-12	-2	0
pork	-60	-11	-1	-93	-16	1	-7	-1	0	-822	-145	-19	-134	-19	-3
poultry	-29	-4	-1	-100	-18	-6	-6	-1	0	-791	-164	-26	-134	-24	-4
eggs	-18	-2	0	-57	-8	0	-4	0	0	-447	-70	-6	-72	-9	-1
milk	-66	-17	24	-84	-26	20	-6	-1	0	-499	-120	22	-124	-25	3
shellfish	0	0	0	-2	0	0	0	0	0	-17	0	-1	-4	0	0
fish (freshwater)	-1	0	0	-15	0	-2	-1	0	0	-126	-2	-6	-23	0	-1
fish (demersal)	0	0	0	-1	0	0	0	0	0	-7	-1	0	-1	0	0

Food group	GHG emissions (MtCO2-eq)			Cropland use (M km2)			Freshwater use (km3)			Nitrogen application (GgN)			Phosphorus application (GgP)		
	Global	HIC	LIC	Global	HIC	LIC	Global	HIC	LIC	Global	HIC	LIC	Global	HIC	LIC
total	-3970	-854	-383	-869	-448	188	-162	-17	42	-17765	-4327	-282	-2181	-629	81
wheat	-30	2	-3	-393	20	-54	-80	-1	-9	-4167	149	-290	-616	18	-41
rice	-172	-1	-29	-495	-4	-123	-151	-1	-22	-5269	-26	-945	-634	-7	-119
maize	-11	0	-5	-136	1	-62	-7	0	0	-969	12	-256	-113	1	-32
other grains	-4	0	-2	-275	1	-107	-6	0	0	-481	7	-78	-80	1	-11
roots	-11	1	-4	-118	2	-33	-8	0	-2	-685	26	-44	-107	5	-6
legumes	33	4	5	610	68	127	67	8	11						
soybeans	8	1	1	225	29	52	11	1	4	229	13	31	284	36	52
nuts & seeds	24	3	11	308	37	77	30	3	4	560	91	211	89	10	26
vegetables	51	4	9	249	16	127	54	2	25	2524	211	821	390	44	126
fruits (temperate)	7	1	1	112	8	34	41	2	20	853	65	129	114	11	17
fruits (tropical)	10	0	2	111	4	36	51	2	10	1101	49	162	154	8	23
fruits (starchy)	2	0	-1	-13	1	-16	7	0	2	178	8	-28	33	1	-4
sugar	-22	-5	0	-159	-24	-5	-126	-6	-3	-2109	-216	-49	-325	-39	-7
palm oil	-27	-2	-3	-47	-2	-6	0	0	0	-300	-14	-34	-41	-2	-5
vegetable oil	53	2	11	676	16	188	39	1	7	1811	70	350	516	15	100
beef	-2652	-529	-267	-250	-91	-13	-14	-4	-1	-1716	-776	-54	-318	-119	-10
lamb	-543	-91	-81	-74	-15	-5	-6	-1	-1	-337	-85	-18	-54	-13	-2
pork	-328	-107	-17	-540	-193	-24	-32	-9	-2	-4364	-1614	-177	-678	-221	-24
poultry	-81	-28	-1	-321	-130	-9	-18	-6	-1	-2503	-1110	-36	-424	-168	-5
eggs	-62	-13	0	-193	-53	-1	-13	-3	0	-1590	-510	-5	-245	-69	-1
milk	-216	-98	-10	-212	-143	-2	-10	-7	0	-947	-694	-8	-218	-145	-1
shellfish	0	0	0	4	1	0	0	0	0	22	4	3	5	1	1
fish (freshwater)	3	0	0	62	1	6	7	0	1	387	10	35	86	2	6
fish (demersal)	0	0	0	1	0	0	0	0	0	5	2	0	1	0	0

Supplementary Table 18. Coefficients of association between health and environmental impacts in 2010 (upper panel) and 2050 (lower panel) calculated by dividing the percentage changes in environmental impacts by the percentage changes in premature mortality.

Region	Domain	Diet scenario											
		FLX	PSC	VEG	VGN	ani-25	ani-50	ani-75	ani-100	kcal-25	kcal-50	kcal-75	kcal-100
Global	GHGe	2.38	3.21	3.25	3.49	5.24	6.04	6.69	7.26	0.11	0.19	0.23	0.25
	land	-0.03	0.10	-0.01	0.01	-0.07	0.16	0.26	0.33	-0.28	-0.06	0.03	0.08
	water	0.05	0.02	-0.13	-0.43	-1.21	-1.37	-1.51	-1.64	-0.06	-0.06	-0.07	-0.07
	nitr	0.80	0.79	0.84	0.82	0.30	0.36	0.40	0.44	0.25	0.31	0.34	0.35
	phos	0.54	0.54	0.59	0.63	0.31	0.37	0.42	0.46	0.25	0.31	0.34	0.35
High-income countries	GHGe	3.41	3.60	3.72	3.73	3.81	4.89	5.68	5.98	0.64	0.63	0.63	0.63
	land	1.21	1.38	1.34	1.45	1.23	1.58	1.84	1.93	0.62	0.61	0.61	0.60
	water	0.43	0.38	0.18	-0.21	-1.60	-2.05	-2.38	-2.50	0.69	0.68	0.68	0.68
	nitr	1.68	1.85	1.90	1.96	1.73	2.22	2.57	2.71	0.62	0.61	0.61	0.61
	phos	1.53	1.67	1.69	1.81	1.56	1.99	2.32	2.44	0.55	0.54	0.54	0.54
Upper middle-income countries	GHGe	2.79	3.14	3.24	3.35	4.64	5.38	6.09	6.72	0.46	0.57	0.75	0.74
	land	0.62	0.81	0.70	0.69	0.58	0.68	0.76	0.84	0.42	0.52	0.67	0.67
	water	0.35	0.26	0.07	-0.34	-1.66	-1.93	-2.18	-2.41	0.49	0.61	0.79	0.79
	nitr	0.95	1.11	1.14	1.13	0.99	1.15	1.30	1.43	0.46	0.57	0.74	0.74
	phos	0.72	0.86	0.88	0.99	1.10	1.28	1.45	1.60	0.54	0.68	0.88	0.88
Lower middle-income countries	GHGe	1.91	3.05	3.06	3.31	5.12	5.55	5.98	6.48	0.17	0.20	0.21	0.21
	land	-0.39	-0.29	-0.35	-0.36	-0.12	-0.13	-0.14	-0.15	0.16	0.19	0.19	0.20
	water	0.24	0.21	0.10	-0.18	-0.88	-0.95	-1.03	-1.11	-0.17	-0.20	-0.20	-0.21
	nitr	0.64	0.54	0.62	0.56	-0.42	-0.45	-0.49	-0.53	0.25	0.29	0.30	0.31
	phos	0.37	0.28	0.37	0.36	-0.39	-0.43	-0.46	-0.50	0.21	0.24	0.24	0.25
Low-income countries	GHGe	1.29	2.95	2.94	3.74	11.39	14.97	17.59	19.21	-1.86	-1.39	-1.23	-1.15
	land	-1.21	-1.11	-1.38	-1.38	-7.76	-4.98	-4.06	-3.51	-3.71	-2.27	-1.76	-1.50
	water	-2.16	-2.24	-2.53	-3.07	-4.90	-5.71	-6.45	-6.92	-0.80	-0.79	-0.80	-0.80
	nitr	-0.40	-0.40	-0.44	-0.41	-1.76	-1.17	-0.99	-0.88	-0.86	-0.55	-0.44	-0.39
	phos	-1.19	-1.20	-1.23	-1.20	-2.22	-1.72	-1.61	-1.55	-0.83	-0.52	-0.41	-0.35

Region	Domain	Diet scenario											
		FLX	PSC	VEG	VGN	ani-25	ani-50	ani-75	ani-100	kcal-25	kcal-50	kcal-75	kcal-100
Global	GHGe	3.10	3.94	4.05	4.10	5.57	6.55	7.32	7.85	1.10	1.39	1.53	1.63
	land	0.76	0.85	0.85	0.81	-0.15	0.23	0.40	0.51	0.83	1.29	1.49	1.61
	water	0.97	0.87	0.82	0.51	-1.04	-1.12	-1.21	-1.27	1.44	1.42	1.46	1.51
	nitr	1.43	1.41	1.51	1.37	0.21	0.35	0.43	0.48	1.67	1.71	1.78	1.85
	phos	1.12	1.08	1.21	1.12	0.10	0.25	0.32	0.37	1.63	1.70	1.78	1.85
High-income countries	GHGe	3.50	3.68	3.83	3.80	4.26	5.18	5.75	6.01	0.95	0.93	0.95	0.93
	land	1.36	1.49	1.51	1.63	1.44	1.75	1.94	2.03	0.95	0.93	0.94	0.93
	water	0.73	0.66	0.54	0.22	-1.36	-1.66	-1.84	-1.92	1.01	0.99	1.01	0.99
	nitr	1.74	1.87	1.95	1.99	1.80	2.19	2.42	2.53	0.93	0.91	0.92	0.91
	phos	1.61	1.69	1.76	1.85	1.64	2.00	2.22	2.32	0.87	0.86	0.87	0.86
Upper middle-income countries	GHGe	3.04	3.31	3.44	3.45	4.66	5.55	6.33	6.53	1.48	1.41	1.52	1.50
	land	1.01	1.12	1.09	1.05	0.64	0.77	0.87	0.90	1.37	1.31	1.42	1.39
	water	0.87	0.78	0.67	0.35	-1.26	-1.50	-1.71	-1.77	1.42	1.35	1.46	1.44
	nitr	1.37	1.46	1.54	1.51	1.20	1.42	1.63	1.68	1.40	1.34	1.45	1.42
	phos	1.11	1.16	1.24	1.28	1.07	1.27	1.45	1.50	1.52	1.45	1.57	1.54
Lower middle-income countries	GHGe	2.77	3.96	4.10	4.08	5.80	6.21	6.72	7.15	2.87	2.39	2.40	2.45
	land	0.70	0.74	0.82	0.74	0.35	0.37	0.41	0.43	2.94	2.44	2.45	2.50
	water	1.28	1.16	1.15	0.80	-0.59	-0.63	-0.68	-0.73	2.32	1.93	1.93	1.98
	nitr	1.45	1.32	1.48	1.21	-0.29	-0.31	-0.33	-0.36	3.07	2.55	2.56	2.62
	phos	1.15	1.01	1.20	0.99	-0.33	-0.35	-0.38	-0.40	3.07	2.55	2.56	2.62
Low-income countries	GHGe	2.96	4.15	4.13	4.66	5.58	9.45	11.78	13.70	-2.00	-0.50	0.05	0.34
	land	0.06	0.21	0.01	-0.01	-5.57	-3.62	-2.86	-2.48	-2.82	-0.77	-0.01	0.39
	water	-0.43	-0.50	-0.67	-1.04	-3.86	-4.06	-4.36	-4.70	-0.22	0.29	0.47	0.59
	nitr	0.79	0.91	0.90	0.98	-2.05	-0.85	-0.31	0.02	-0.91	0.19	0.57	0.81
	phos	-0.18	-0.10	-0.07	-0.01	-3.43	-1.94	-1.33	-0.98	-1.68	-0.21	0.31	0.61

Supplementary Figure 2. Change in environmental impacts (%) using a global meta-analysis of life-cycle analyses for GHG emissions instead of country-specific data on methane and nitrous oxide emissions.

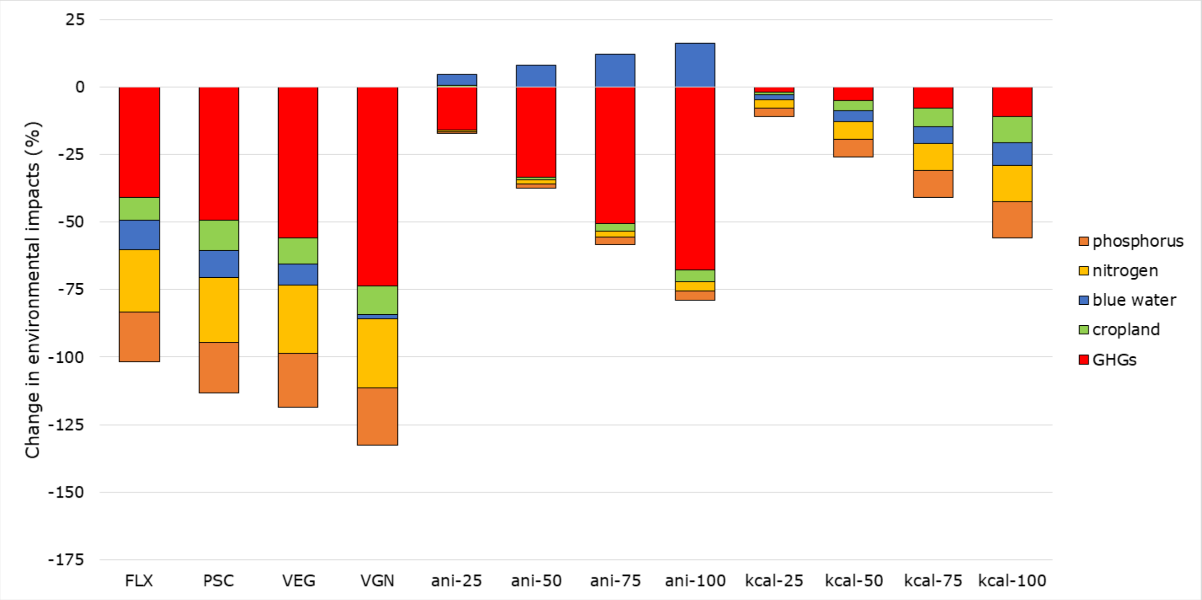

References

1 Robinson S, Mason-D'Croz D, Islam S, *et al.* The International Model for Policy Analysis of Agricultural Commodities and Trade (IMPACT) -- Model description for version 3. 2015.

2 Gustavsson J, Cederberg C, Sonesson U, Van Otterdijk R, Meybeck A. Global food losses and food waste: extent, causes and prevention. FAO Rome, 2011.

3 Alexandratos N, Bruinsma J. World agriculture towards 2030/2050: the 2012 revision. Rome: FAO, 2012.

4 Valin H, Sands RD, van der Mensbrugghe D, *et al.* The future of food demand: understanding differences in global economic models. *Agric Econ* 2014; **45**: 51–67.

5 Cover TM, Thomas JA. Elements of information theory. John Wiley & Sons, 2012.

6 Ludwig DS, Willett WC. Three daily servings of reduced-fat milk: an evidence-based recommendation? *JAMA Pediatr* 2013; **167**: 788–9.

7 Mozaffarian D, Appel LJ, Horn L Van. Components of a Cardioprotective Diet. *Circulation* 2011; **123**: 2870–91.

8 Katz DL, Meller S. Can we say what diet is best for health? *Annu Rev Public Health* 2014; **35**: 83–103.

9 Health USD of, Services H. Dietary guidelines for Americans 2015-2020. Skyhorse Publishing Inc., 2017.

10 Haddad EH, Tanzman JS. What do vegetarians in the United States eat? *Am J Clin Nutr* 2003; **78**: 626S–632S.

11 Bradbury KE, Tong TYN, Key TJ. Dietary Intake of High-Protein Foods and Other Major Foods in Meat-Eaters, Poultry-Eaters, Fish-Eaters, Vegetarians, and Vegans in UK Biobank. *Nutrients* 2017; **9**. DOI:10.3390/nu9121317.

12 Smith MR, Micha R, Golden CD, Mozaffarian D, Myers SS. Global Expanded Nutrient Supply (GENuS) Model: A New Method for Estimating the Global Dietary Supply of Nutrients. *PLoS One* 2016; **11**: e0146976.

13 WHO. Trace elements in human nutrition and health. World Health Organization, 1996.

14 WHO. Diet, nutrition and the prevention of chronic diseases: Report of the joint WHO/FAO expert consultation. Geneva, Switzerland: WHO, 2003.

15 Wang H, Naghavi M, Allen C, *et al.* Global, regional, and national life expectancy, all-cause mortality, and cause-specific mortality for 249 causes of death, 1980–2015: a systematic analysis for the Global Burden of Disease Study 2015. *Lancet* 2016; **388**: 1459–544.

16 DeSA UN. World population prospects: the 2012 revision. *Popul Div Dep Econ Soc Aff United Nations Secr New York* 2013.

17 WHO. Human energy requirements: Report of a Joint FAO/WHO/UNU Expert Consultation, Rome, Italy, 17-24 October 2001. Geneva, Switzerland: WHO, 2004.

18 Beal T, Massiot E, Arsenault JE, Smith MR, Hijmans RJ. Global trends in dietary micronutrient supplies and estimated prevalence of inadequate intakes. *PLoS One* 2017; **12**: e0175554.

19 Murray CJL, Ezzati M, Lopez AD, Rodgers A, Vander Hoorn S. Comparative quantification of health risks: conceptual framework and methodological issues. *Popul Health Metr* 2003; **1**: 1.

20 Lim SS, Vos T, Flaxman AD, *et al.* A comparative risk assessment of burden of disease and injury attributable to 67 risk factors and risk factor clusters in 21 regions, 1990–2010: a systematic analysis for the Global Burden of Disease Study 2010. *Lancet* 2012; **380**: 2224–60.

21 Forouzanfar MH, Alexander L, Anderson HR, *et al.* Global, regional, and national comparative risk assessment of 79 behavioural, environmental and occupational, and metabolic risks or clusters of risks in 188 countries, 1990–2013: a systematic analysis for the Global Burden of Disease Study 2013. *Lancet* 2015; **386**: 2287–323.
22 Murray CJL, Ezzati M, Flaxman AD, *et al.* GBD 2010: design, definitions, and metrics. *Lancet* 2012; **380**: 2063–6.
23 Lozano R, Naghavi M, Foreman K, *et al.* Global and regional mortality from 235 causes of death for 20 age groups in 1990 and 2010: a systematic analysis for the Global Burden of Disease Study 2010. *Lancet* 2012; **380**: 2095–128.
24 Afshin A, Micha R, Khatibzadeh S, Mozaffarian D. Consumption of nuts and legumes and risk of incident ischemic heart disease, stroke, and diabetes: a systematic review and meta-analysis. *Am J Clin Nutr* 2014; : ajcn.076901.
25 Aune D, Keum N, Giovannucci E, *et al.* Nut consumption and risk of cardiovascular disease, total cancer, all-cause and cause-specific mortality: a systematic review and dose-response meta-analysis of prospective studies. *BMC Med* 2016; **14**: 207.
26 Aune D, Giovannucci E, Boffetta P, *et al.* Fruit and vegetable intake and the risk of cardiovascular disease, total cancer and all-cause mortality–a systematic review and dose-response meta-analysis of prospective studies. *Int J Epidemiol* 2016; published online March 18. http://eprints.whiterose.ac.uk/106013/.
27 Chan DSM, Lau R, Aune D, *et al.* Red and processed meat and colorectal cancer incidence: meta-analysis of prospective studies. *PLoS One* 2011; **6**: e20456.
28 Chen G-C, Lv D-B, Pang Z, Liu Q-F. Red and processed meat consumption and risk of stroke: a meta-analysis of prospective cohort studies. *Eur J Clin Nutr* 2013; **67**: 91–5.
29 Feskens EJM, Sluik D, van Woudenbergh GJ. Meat consumption, diabetes, and its complications. *Curr Diab Rep* 2013; **13**: 298–306.
30 Micha R, Michas G, Mozaffarian D. Unprocessed red and processed meats and risk of coronary artery disease and type 2 diabetes--an updated review of the evidence. *Curr Atheroscler Rep* 2012; **14**: 515–24.
31 Zheng J, Huang T, Yu Y, Hu X, Yang B, Li D. Fish consumption and CHD mortality: an updated meta-analysis of seventeen cohort studies. *Public Health Nutr* 2012; **15**: 725–37.
32 Collaboration PS, Whitlock G, Lewington S, *et al.* Body-mass index and cause-specific mortality in 900 000 adults: collaborative analyses of 57 prospective studies. *Lancet* 2009; **373**: 1083–96.
33 Berrington de Gonzalez A, Hartge P, Cerhan JR, *et al.* Body-Mass Index and Mortality among 1.46 Million White Adults. *N Engl J Med* 2010; **363**: 2211–9.
34 Singh GM, Danaei G, Farzadfar F, *et al.* The Age-Specific Quantitative Effects of Metabolic Risk Factors on Cardiovascular Diseases and Diabetes: A Pooled Analysis. *PLoS One* 2013; **8**: e65174.
35 Micha R, Shulkin ML, Peñalvo JL, *et al.* Etiologic effects and optimal intakes of foods and nutrients for risk of cardiovascular diseases and diabetes: Systematic reviews and meta-analyses from the Nutrition and Chronic Diseases Expert Group (NutriCoDE). *PLoS One* 2017; **12**: e0175149.
36 Micha R, Wallace SK, Mozaffarian D. Red and processed meat consumption and risk of incident coronary heart disease, stroke, and diabetes mellitus: a systematic review and meta-analysis. *Circulation* 2010; **121**: 2271–83.
37 Kaluza J, Wolk A, Larsson SC. Red meat consumption and risk of stroke: a meta-analysis of prospective studies. *Stroke* 2012; **43**: 2556–60.
38 Yang C, Pan L, Sun C, Xi Y, Wang L, Li D. Red Meat Consumption and the Risk of Stroke: A Dose-Response Meta-analysis of Prospective Cohort Studies. *J Stroke*

Cerebrovasc Dis Off J Natl Stroke Assoc 2016; **25**: 1177–86.
39 Abete I, Romaguera D, Vieira AR, Lopez de Munain A, Norat T. Association between total, processed, red and white meat consumption and all-cause, CVD and IHD mortality: a meta-analysis of cohort studies. *Br J Nutr* 2014; **112**: 762–75.
40 Wang X, Lin X, Ouyang YY, *et al.* Red and processed meat consumption and mortality: dose-response meta-analysis of prospective cohort studies. *Public Health Nutr* 2016; **19**: 893–905.
41 Aune D, Chan DSM, Vieira AR, *et al.* Red and processed meat intake and risk of colorectal adenomas: a systematic review and meta-analysis of epidemiological studies. *Cancer causes Control CCC* 2013; **24**: 611–27.
42 Bouvard V, Loomis D, Guyton KZ, *et al.* Carcinogenicity of consumption of red and processed meat. *Lancet Oncol* 2015; **16**: 1599–600.
43 Larsson SC, Wolk A. Meat consumption and risk of colorectal cancer: a meta-analysis of prospective studies. *Int J Cancer* 2006; **119**: 2657–64.
44 WCRF/AICR. Food, Nutrition, Physical Activity, and the Prevention of Cancer: A Global Perspective. Washington, DC, USA: AICR, 2007.
45 Larsson SC, Orsini N. Red meat and processed meat consumption and all-cause mortality: a meta-analysis. *Am J Epidemiol* 2013; : kwt261.
46 Wolk A. Potential health hazards of eating red meat. *J Intern Med* 2017; **281**: 106–22.
47 Chen G-C, Lv D-B, Pang Z, Liu Q-F. Red and processed meat consumption and risk of stroke: a meta-analysis of prospective cohort studies. *Eur J Clin Nutr* 2013; **67**: 91–5.
48 Bernstein AM, Pan A, Rexrode KM, *et al.* Dietary protein sources and the risk of stroke in men and women. *Stroke* 2011; : STROKEAHA-111.
49 Bernstein AM, Sun Q, Hu FB, Stampfer MJ, Manson JE, Willett WC. Major dietary protein sources and risk of coronary heart disease in women. *Circulation* 2010; **122**: 876–83.
50 Pan A, Sun Q, Bernstein AM, *et al.* Red meat consumption and mortality: results from 2 prospective cohort studies. *Arch Intern Med* 2012; **172**: 555–63.
51 Pan A, Sun Q, Bernstein AM, *et al.* Red meat consumption and risk of type 2 diabetes: 3 cohorts of US adults and an updated meta-analysis. *Am J Clin Nutr* 2011; **94**: 1088–96.
52 Larsson SC, Crippa A, Orsini N, Wolk A, Michaëlsson K. Milk Consumption and Mortality from All Causes, Cardiovascular Disease, and Cancer: A Systematic Review and Meta-Analysis. *Nutrients* 2015; **7**: 7749–63.
53 Mullie P, Pizot C, Autier P. Daily milk consumption and all-cause mortality, coronary heart disease and stroke: a systematic review and meta-analysis of observational cohort studies. *BMC Public Health* 2016; **16**: 1236.
54 Soedamah-Muthu SS, Ding EL, Al-Delaimy WK, *et al.* Milk and dairy consumption and incidence of cardiovascular diseases and all-cause mortality: dose-response meta-analysis of prospective cohort studies. *Am J Clin Nutr* 2011; **93**: 158–71.
55 Aune D, Lau R, Chan DSM, *et al.* Dairy products and colorectal cancer risk: a systematic review and meta-analysis of cohort studies. *Ann Oncol Off J Eur Soc Med Oncol* 2012; **23**: 37–45.
56 Aune D, Norat T, Romundstad P, Vatten LJ. Dairy products and the risk of type 2 diabetes: a systematic review and dose-response meta-analysis of cohort studies. *Am J Clin Nutr* 2013; **98**: 1066–83.
57 Aune D, Navarro Rosenblatt DA, Chan DSM, *et al.* Dairy products, calcium, and prostate cancer risk: a systematic review and meta-analysis of cohort studies. *Am J Clin Nutr* 2015; **101**: 87–117.
58 Lu W, Chen H, Niu Y, Wu H, Xia D, Wu Y. Dairy products intake and cancer

mortality risk: a meta-analysis of 11 population-based cohort studies. *Nutr J* 2016; **15**: 91.
59 Alexander DD, Bylsma LC, Vargas AJ, *et al.* Dairy consumption and CVD: a systematic review and meta-analysis. *Br J Nutr* 2016; **115**: 737–50.
60 de Goede J, Soedamah-Muthu SS, Pan A, Gijsbers L, Geleijnse JM. Dairy Consumption and Risk of Stroke: A Systematic Review and Updated Dose-Response Meta-Analysis of Prospective Cohort Studies. *J Am Heart Assoc* 2016; **5**. DOI:10.1161/JAHA.115.002787.
61 Gijsbers L, Ding EL, Malik VS, de Goede J, Geleijnse JM, Soedamah-Muthu SS. Consumption of dairy foods and diabetes incidence: a dose-response meta-analysis of observational studies. *Am J Clin Nutr* 2016; **103**: 1111–24.
62 Bolland MJ, Leung W, Tai V, *et al.* Calcium intake and risk of fracture: systematic review. *BMJ* 2015; **351**: h4580.
63 Bischoff-Ferrari HA, Dawson-Hughes B, Baron JA, *et al.* Milk intake and risk of hip fracture in men and women: a meta-analysis of prospective cohort studies. *J Bone Miner Res Off J Am Soc Bone Miner Res* 2011; **26**: 833–9.
64 Tai V, Leung W, Grey A, Reid IR, Bolland MJ. Calcium intake and bone mineral density: systematic review and meta-analysis. *BMJ* 2015; **351**: h4183.
65 Kretchmer N. Lactose and lactase--a historical perspective. *Gastroenterology* 1971; **61**: 805–13.
66 Lomer MCE, Parkes GC, Sanderson JD. Review article: lactose intolerance in clinical practice--myths and realities. *Aliment Pharmacol Ther* 2008; **27**: 93–103.
67 Scrimshaw NS, Murray EB. The acceptability of milk and milk products in populations with a high prevalence of lactose intolerance. *Am J Clin Nutr* 1988; **48**: 1079–159.
68 Deng Y, Misselwitz B, Dai N, Fox M. Lactose Intolerance in Adults: Biological Mechanism and Dietary Management. *Nutrients* 2015; **7**: 8020–35.
69 He K, Song Y, Daviglus ML, *et al.* Accumulated evidence on fish consumption and coronary heart disease mortality: a meta-analysis of cohort studies. *Circulation* 2004; **109**: 2705–11.
70 Larsson SC, Orsini N. Fish consumption and the risk of stroke: a dose-response meta-analysis. *Stroke* 2011; **42**: 3621–3.
71 Xun P, Qin B, Song Y, *et al.* Fish consumption and risk of stroke and its subtypes: accumulative evidence from a meta-analysis of prospective cohort studies. *Eur J Clin Nutr* 2012; **66**: 1199–207.
72 Zhao L-G, Sun J-W, Yang Y, Ma X, Wang Y-Y, Xiang Y-B. Fish consumption and all-cause mortality: a meta-analysis of cohort studies. *Eur J Clin Nutr* 2016; **70**: 155–61.
73 Wallin A, Di Giuseppe D, Orsini N, Patel PS, Forouhi NG, Wolk A. Fish consumption, dietary long-chain n-3 fatty acids, and risk of type 2 diabetes: systematic review and meta-analysis of prospective studies. *Diabetes Care* 2012; **35**: 918–29.
74 Zhang M, Picard-Deland E, Marette A. Fish and marine omega-3 polyunsatured Fatty Acid consumption and incidence of type 2 diabetes: a systematic review and meta-analysis. *Int J Endocrinol* 2013; **2013**: 501015.
75 Del Gobbo LC, Imamura F, Aslibekyan S, *et al.* ω-3 Polyunsaturated Fatty Acid Biomarkers and Coronary Heart Disease: Pooling Project of 19 Cohort Studies. *JAMA Intern Med* 2016; **176**: 1155–66.
76 Afshin A, Micha R, Khatibzadeh S, Mozaffarian D. Consumption of nuts and legumes and risk of incident ischemic heart disease, stroke, and diabetes: a systematic review and meta-analysis. *Am J Clin Nutr* 2014; **100**: 278–88.

77 Luo C, Zhang Y, Ding Y, *et al.* Nut consumption and risk of type 2 diabetes, cardiovascular disease, and all-cause mortality: a systematic review and meta-analysis. *Am J Clin Nutr* 2014; **100**: 256–69.
78 Mayhew AJ, de Souza RJ, Meyre D, Anand SS, Mente A. A systematic review and meta-analysis of nut consumption and incident risk of CVD and all-cause mortality. *Br J Nutr* 2016; **115**: 212–25.
79 Grosso G, Yang J, Marventano S, Micek A, Galvano F, Kales SN. Nut consumption on all-cause, cardiovascular, and cancer mortality risk: a systematic review and meta-analysis of epidemiologic studies. *Am J Clin Nutr* 2015; **101**: 783–93.
80 Shi ZQ, Tang JJ, Wu H, Xie CY, He ZZ. Consumption of nuts and legumes and risk of stroke: a meta-analysis of prospective cohort studies. *Nutr Metab Cardiovasc Dis NMCD* 2014; **24**: 1262–71.
81 Zhou D, Yu H, He F, *et al.* Nut consumption in relation to cardiovascular disease risk and type 2 diabetes: a systematic review and meta-analysis of prospective studies. *Am J Clin Nutr* 2014; **100**: 270–7.
82 Zhu B, Sun Y, Qi L, Zhong R, Miao X. Dietary legume consumption reduces risk of colorectal cancer: evidence from a meta-analysis of cohort studies. *Sci Rep* 2015; **5**: 8797.
83 Dauchet L, Amouyel P, Hercberg S, Dallongeville J. Fruit and vegetable consumption and risk of coronary heart disease: a meta-analysis of cohort studies. *J Nutr* 2006; **136**: 2588–93.
84 Gan Y, Tong X, Li L, *et al.* Consumption of fruit and vegetable and risk of coronary heart disease: A meta-analysis of prospective cohort studies. *Int J Cardiol* 2015; **183**: 129–37.
85 He FJ, Nowson CA, Lucas M, MacGregor GA. Increased consumption of fruit and vegetables is related to a reduced risk of coronary heart disease: meta-analysis of cohort studies. *J Hum Hypertens* 2007; **21**: 717–28.
86 Dauchet L, Amouyel P, Dallongeville J. Fruit and vegetable consumption and risk of stroke: a meta-analysis of cohort studies. *Neurology* 2005; **65**: 1193–7.
87 Hu D, Huang J, Wang Y, Zhang D, Qu Y. Fruits and Vegetables Consumption and Risk of Stroke. *Stroke* 2014; **45**: 1613–9.
88 Li M, Fan Y, Zhang X, Hou W, Tang Z. Fruit and vegetable intake and risk of type 2 diabetes mellitus: meta-analysis of prospective cohort studies. *BMJ Open* 2014; **4**: e005497.
89 Wu Y, Zhang D, Jiang X, Jiang W. Fruit and vegetable consumption and risk of type 2 diabetes mellitus: a dose-response meta-analysis of prospective cohort studies. *Nutr Metab Cardiovasc Dis NMCD* 2015; **25**: 140–7.
90 Wang X, Ouyang Y, Liu J, *et al.* Fruit and vegetable consumption and mortality from all causes, cardiovascular disease, and cancer: systematic review and dose-response meta-analysis of prospective cohort studies. *BMJ* 2014; **349**: g4490.
91 Wang X, Ouyang Y, Liu J, *et al.* Fruit and vegetable consumption and mortality from all causes, cardiovascular disease, and cancer: systematic review and dose-response meta-analysis of prospective cohort studies. *BMJ* 2014; **349**: g4490–g4490.
92 Vieira AR, Abar L, Vingeliene S, *et al.* Fruits, vegetables and lung cancer risk: a systematic review and meta-analysis. *Ann Oncol* 2015; : mdv381.
93 Bjelakovic G, Nikolova D, Gluud LL, Simonetti RG, Gluud C. Antioxidant supplements for prevention of mortality in healthy participants and patients with various diseases. *Cochrane Database Syst Rev* 2012; : CD007176.
94 Schwingshackl L, Boeing H, Stelmach-Mardas M, *et al.* Dietary Supplements and Risk of Cause-Specific Death, Cardiovascular Disease, and Cancer: A Systematic Review

and Meta-Analysis of Primary Prevention Trials. *Adv Nutr* 2017; **8**: 27–39.
95 Liu RH. Health-Promoting Components of Fruits and Vegetables in the Diet. *Adv Nutr An Int Rev J* 2013; **4**: 384S–392S.
96 Liu RH. Dietary Bioactive Compounds and Their Health Implications. *J Food Sci* 2013; **78**: A18–25.
97 Borgi L, Rimm EB, Willett WC, Forman JP. Potato intake and incidence of hypertension: results from three prospective US cohort studies. *BMJ* 2016; **353**: i2351.
98 King JC, Slavin JL. White potatoes, human health, and dietary guidance. *Adv Nutr* 2013; **4**: 393S–401S.
99 Muraki I, Rimm EB, Willett WC, Manson JE, Hu FB, Sun Q. Potato Consumption and Risk of Type 2 Diabetes: Results From Three Prospective Cohort Studies. *Diabetes Care* 2016; **39**: 376–84.
100 Aune D, Keum N, Giovannucci E, *et al.* Whole grain consumption and risk of cardiovascular disease, cancer, and all cause and cause specific mortality: systematic review and dose-response meta-analysis of prospective studies. *BMJ* 2016; **353**: i2716.
101 Zong G, Gao A, Hu FB, Sun Q. Whole Grain Intake and Mortality From All Causes, Cardiovascular Disease, and Cancer: A Meta-Analysis of Prospective Cohort Studies. *Circulation* 2016; **133**: 2370–80.
102 Ye EQ, Chacko SA, Chou EL, Kugizaki M, Liu S. Greater whole-grain intake is associated with lower risk of type 2 diabetes, cardiovascular disease, and weight gain. *J Nutr* 2012; **142**: 1304–13.
103 Aune D, Norat T, Romundstad P, Vatten LJ. Whole grain and refined grain consumption and the risk of type 2 diabetes: a systematic review and dose–response meta-analysis of cohort studies. *Eur J Epidemiol* 2013; **28**: 845–58.
104 Chen J, Huang Q, Shi W, Yang L, Chen J, Lan Q. Meta-Analysis of the Association Between Whole and Refined Grain Consumption and Stroke Risk Based on Prospective Cohort Studies. *Asia-Pacific J Public Heal* 2016; **28**: 563–75.
105 Hu EA, Pan A, Malik V, Sun Q. White rice consumption and risk of type 2 diabetes: meta-analysis and systematic review. *BMJ* 2012; **344**: e1454.
106 Malik VS, Pan A, Willett WC, Hu FB. Sugar-sweetened beverages and weight gain in children and adults: a systematic review and meta-analysis. *Am J Clin Nutr* 2013; **98**: 1084–102.
107 Te Morenga L, Mallard S, Mann J. Dietary sugars and body weight: systematic review and meta-analyses of randomised controlled trials and cohort studies. *BMJ* 2012; **346**: e7492.
108 Malik VS, Popkin BM, Bray GA, Després J-P, Willett WC, Hu FB. Sugar-sweetened beverages and risk of metabolic syndrome and type 2 diabetes: a meta-analysis. *Diabetes Care* 2010; **33**: 2477–83.
109 Te Morenga LA, Howatson AJ, Jones RM, Mann J. Dietary sugars and cardiometabolic risk: systematic review and meta-analyses of randomized controlled trials of the effects on blood pressure and lipids. *Am J Clin Nutr* 2014; **100**: 65–79.
110 Imamura F, O'Connor L, Ye Z, *et al.* Consumption of sugar sweetened beverages, artificially sweetened beverages, and fruit juice and incidence of type 2 diabetes: systematic review, meta-analysis, and estimation of population attributable fraction. *BMJ* 2015; **351**: h3576.
111 Malik VS, Hu FB. Fructose and Cardiometabolic Health: What the Evidence From Sugar-Sweetened Beverages Tells Us. *J Am Coll Cardiol* 2015; **66**: 1615–24.
112 WC W, JE M, MJ S, al et. Weight, weight change, and coronary heart disease in women: Risk within the 'normal' weight range. *JAMA* 1995; **273**: 461–5.
113 Collaboration APCS. Body mass index and cardiovascular disease in the Asia-Pacific

Region: an overview of 33 cohorts involving 310 000 participants. *Int J Epidemiol* 2004; **33**: 751–8.
114 Song Y-M, Sung J, Smith GD, Ebrahim S. Body Mass Index and Ischemic and Hemorrhagic Stroke A Prospective Study in Korean Men. *Stroke* 2004; **35**: 831–6.
115 KM R, CH H, WC W, al et. A prospective study of body mass index, weight change, and risk of stroke in women. *JAMA* 1997; **277**: 1539–45.
116 Calle EE, Rodriguez C, Walker-Thurmond K, Thun MJ. Overweight, obesity, and mortality from cancer in a prospectively studied cohort of U.S. adults. *N Engl J Med* 2003; **348**: 1625–38.
117 Reeves GK, Pirie K, Beral V, *et al.* Cancer incidence and mortality in relation to body mass index in the Million Women Study: cohort study. *BMJ* 2007; **335**: 1134.
118 Parr CL, Batty GD, Lam TH, *et al.* Body-mass index and cancer mortality in the Asia-Pacific Cohort Studies Collaboration: pooled analyses of 424,519 participants. *Lancet Oncol* 2010; **11**: 741–52.
119 Calle EE, Kaaks R. Overweight, obesity and cancer: epidemiological evidence and proposed mechanisms. *Nat Rev Cancer* 2004; **4**: 579–91.
120 Willett WC, Dietz WH, Colditz GA. Guidelines for healthy weight. *N Engl J Med* 1999; **341**: 427–34.
121 Chiolero A, Kaufman JS. Metabolic mediators of body-mass index and cardiovascular risk. *Lancet* 2014; **383**: 2042.
122 Yusuf S, Hawken S, Ounpuu S, *et al.* Effect of potentially modifiable risk factors associated with myocardial infarction in 52 countries (the INTERHEART study): case-control study. *Lancet* 2004; **364**: 937–52.
123 Khaw K-T, Wareham N, Bingham S, Welch A, Luben R, Day N. Combined Impact of Health Behaviours and Mortality in Men and Women: The EPIC-Norfolk Prospective Population Study. *PLoS Med* 2008; **5**: e12.
124 Dam RM van, Li T, Spiegelman D, Franco OH, Hu FB. Combined Impact of Lifestyle Factors on Mortality: Prospective Cohort Study in US Women. *BMJ Br Med J* 2008; **337**: 742–5.
125 Huxley RR, Ansary-Moghaddam A, Clifton P, Czernichow S, Parr CL, Woodward M. The impact of dietary and lifestyle risk factors on risk of colorectal cancer: a quantitative overview of the epidemiological evidence. *Int J Cancer* 2009; **125**: 171–80.
126 Nechuta SJ, Shu X-O, Li H-L, *et al.* Combined impact of lifestyle-related factors on total and cause-specific mortality among Chinese women: prospective cohort study. *PLoS Med* 2010; **7**. DOI:10.1371/journal.pmed.1000339.
127 Organization WH. Physical status: the use and interpretation of anthropometry. Report of a WHO Expert Committee. Technical Report Series No. 854. Geneva, Switzerland: World Health Organization, 1995 http://www.who.int/childgrowth/publications/physical_status/en/.
128 Springmann M, Clark M, Mason-D'Croz D, *et al.* Options for keeping the food system within environmental limits. *Nature*.
129 Carlson KM, Gerber JS, Mueller ND, *et al.* Greenhouse gas emissions intensity of global croplands. *Nat Clim Chang* 2017; **7**: 63–8.
130 Tubiello FN, Salvatore M, Rossi S, Ferrara A, Fitton N, Smith P. The FAOSTAT database of greenhouse gas emissions from agriculture. *Environ Res Lett* 2013; **8**: 15009.
131 Troell M, Naylor RL, Metian M, *et al.* Does aquaculture add resilience to the global food system? *Proc Natl Acad Sci* 2014; **111**: 13257–63.
132 Chan CY, Tran N, Dao DC, *et al.* Fish to 2050 in the ASEAN Region. WorldFish

Center and Intl Food Policy Res Inst, 2017 https://books.google.co.uk/books?hl=en&lr=&id=5c4kDgAAQBAJ&oi=fnd&pg=PA4&dq=fish+to+2050+in+asean&ots=q56v8tXw8P&sig=s36AmEqIKDNoy6YrwaqL8iMt10k.

133 Rosegrant MW, Sulser TB, Mason-D'Croz D, *et al.* Quantitative foresight modeling to inform the CGIAR research portfolio. Intl Food Policy Res Inst, 2017 https://books.google.co.uk/books?hl=en&lr=&id=VmS1DgAAQBAJ&oi=fnd&pg=PA1&dq=Quantitative+Foresight+Modeling+to+Inform+the+CGIAR+Research+Portfolio&ots=EO1oxLX785&sig=zchO0EKBcoBTTfAgKbfMQdpTWjo.

134 Beach RH, Creason J, Ohrel SB, *et al.* Global mitigation potential and costs of reducing agricultural non-CO2 greenhouse gas emissions through 2030. *J Integr Environ Sci* 2015; **12**: 87–105.

135 Group IW. Technical update on the social cost of carbon for regulatory impact analysis-under executive order 12866. United States Government, 2013.

136 Mueller ND, Gerber JS, Johnston M, Ray DK, Ramankutty N, Foley JA. Closing yield gaps through nutrient and water management. *Nature* 2012; **490**: 254.

137 Heffer P. Assessment of Fertilizer Use by Crop at the Global Level 2010–2010/11. 2013.

138 Sutton MA, Bleeker A, Howard CM, *et al.* Our Nutrient World: The challenge to produce more food and energy with less pollution. Global Overview of Nutrient Management. Centre for Ecology and Hydrology, Edinburgh on behalf of the Global Partnership on Nutrient Management and the International Nit. *Edinburgh behalf Glob Partnersh Nutr Manag Int Nitrogen Initiat* 2013.

139 Cordell D, White S. Life's bottleneck: sustaining the world's phosphorus for a food secure future. *Annu Rev Environ Resour* 2014; **39**: 161–88.

140 Chateau J, Dellink R, Lanzi E, Magne B. Long-term economic growth and environmental pressure: reference scenarios for future global projections. OECD. 2012.

141 KC S, Lutz W. The human core of the shared socioeconomic pathways: Population scenarios by age, sex, and level of education for all countries to 2100. *Glob Environ Chang* 2014.

142 O'Neill BC, Kriegler E, Riahi K, *et al.* A new scenario framework for climate change research: the concept of shared socioeconomic pathways. *Clim Change* 2014; **122**: 387–400.
